# Excellent thermoelectric performance of Bi_2_MO_4_Cl (M = Y, La, and Bi) derived from ultra-low lattice thermal conductivity

**DOI:** 10.1039/d5ta05523g

**Published:** 2025-09-22

**Authors:** Shipeng Bi, Christopher N. Savory, Alexander G. Squires, Dan Han, Kieran B. Spooner, David O. Scanlon

**Affiliations:** a Department of Chemistry, University College London 20 Gordon Street London WC1H 0AJ UK; b Department of Chemistry, Swansea University Singleton Park Swansea SA2 8PP UK; c School of Chemistry, University of Birmingham Edgbaston Birmingham B15 2TT UK d.o.scanlon@bham.ac.uk; d School of Materials Science and Engineering, Jilin University Changchun 130012 China

## Abstract

Layered mixed-anion oxides are considered potential candidates for thermoelectric materials because they typically possess the advantages of oxides (high-temperature stability, low toxicity, and the use of cost-effective elements) and layered mixed-anion compounds (strong phonon anharmonicity and bonding heterogeneity). In this paper, we predicted the thermoelectric performance of environmentally friendly layered mixed-anion oxides Bi_2_MO_4_Cl (M = Y, La, and Bi) using density functional theory (DFT) calculations. The results show that Bi_3_O_4_Cl and Bi_2_LaO_4_Cl exhibit ultra-low average lattice thermal conductivities of less than 0.3 W m^−1^ K^−1^ at 1000 K, which are attributed to the combined effects of heavy atoms, weak ionic bonding, strong phonon anharmonicity, and low structural symmetry. In addition, the weak ionic bonding significantly inhibits out-of-plane heat transfer, resulting in the lattice thermal conductivity in the out-of-plane direction being the lowest compared to other directions. As a result, under dopable conditions, the predicted p-type maximum average *ZT* of Bi_3_O_4_Cl reaches 2.20 at 1000 K, which is superior to the thermoelectric performance of currently known environmentally friendly thermoelectric materials, and the predicted p-type maximum *ZT* of Bi_2_LaO_4_Cl is over 4 in the out-of-plane direction. These results illustrate the potential for the excellent thermoelectric performance of Bi_2_MO_4_Cl (M = Y, La, and Bi), and also highlight the application potential of layered mixed-anion compounds in achieving low lattice thermal conductivity and enhancing thermoelectric performance.

## Abstract 2

Based on DFT calculations, the average *ZT* of Bi_3_O_4_Cl is predicted to reach 2.2 at 1000 K and the out-of-plane *ZT* of Bi_2_LaO_4_Cl to exceed 4, mainly due to their ultra-low lattice thermal conductivity.

## Graphical abstract

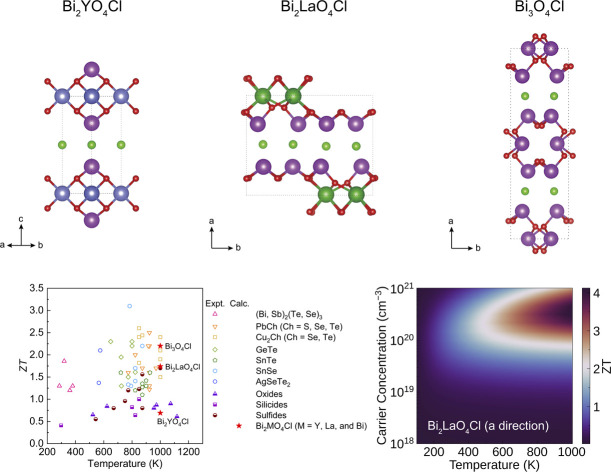

## Introduction

The growing dependence of human civilisation on science and technology has led to an increasing demand for energy, and humanity will have to address the environmental problems caused by this rapidly increasing energy usage.^1^ It is estimated that approximately 50% of global energy is currently wasted as thermal energy.^2^ If this thermal energy can be converted into useful electrical energy by thermoelectric (TE) materials through the Seebeck effect, it could reduce our energy dependence to a great extent.

The performance of TE materials can be evaluated by the dimensionless figure of merit *ZT*:1
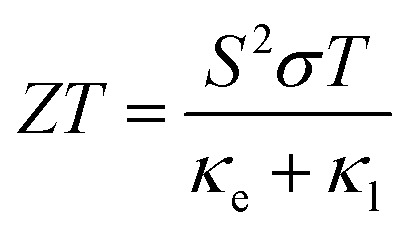
where *S* is the Seebeck coefficient, *σ* is the conductivity, *T* is the absolute temperature, *κ*_e_ and *κ*_l_ are the electronic thermal conductivity and lattice thermal conductivity, respectively. Theoretically, an ideal TE material should have both high electrical conductivity and low thermal conductivity, referred to as the “phonon-glass, electron-crystal” (PGEC), where the lattice conducts heat as in a glass and the electrons conduct as in a crystal.^3^ However, optimising *ZT* is not straightforward because the parameters in eqn (1) are constrained by each other. Low charge carrier concentration and large charge carrier effective mass yield high Seebeck coefficient, but achieving high conductivity requires high charge carrier concentration and small charge carrier effective mass. In addition, according to the Wiedemann–Franz law,^4^ high conductivity inherently implies high electronic thermal conductivity. Therefore, attaining high *ZT* requires a balance between these conflicting parameters. Although researchers have discovered and developed a series of TE materials over the past few decades, few of them are suitable for widespread application due to the lack of high-temperature stability, the inclusion of expensive or toxic elements (such as Te and Pb), or low efficiency.^5–7^

For the next generation of TE materials, high TE conversion efficiency is necessary, and the cost and environmental compatibility of the constituent elements should also be considered. Oxides are considered promising due to their high-temperature stability, low toxicity, and composition of cost-effective elements.^8–10^ However, the *ZT* values of oxide TE materials remain low, partly because their thermal conductivity is still relatively high compared to that of state-of-the-art TE materials PbTe and SnSe.^11–20^ In recent years, layered mixed-anion oxides have garnered significant attention. Leveraging the unique properties conferred by additional anions, these compounds exhibit outstanding characteristics in fields such as electronics, optics, and energy applications.^21^ Layered mixed-anion oxides are also considered highly prospective TE materials. This conclusion is fully supported by theory: for layered mixed-anion oxides, layers are primarily connected by weak ionic bonds, and these weak interactions enhance phonon anharmonicity and hinder out-of-plane phonon heat transport.^21–23^ Besides, the introduction of other anions may cause inhomogeneity in bond strength (bonding heterogeneity), thereby enhancing phonon scattering.^24^ BiCuSeO is a representative layered mixed-anion oxide TE material, receiving widespread attention due to its extremely low intrinsic thermal conductivity.^25^ Its low thermal conductivity originates from a large number of scattering channels and a high Grüneisen parameter.^26–28^ Currently, the *ZT* of BiCuSeO with co-doped metal elements can reach 1.5 at 873 K.^29^

Additionally, many computational studies have also shown that layered mixed-anion oxides have the potential for excellent TE performance. First-principles calculations on the layered mixed-anion oxides LaZnOP and LaZnOAs revealed high out-of-plane p-type *ZT* values of 2.10 and 1.92 at 1000 K, respectively.^30^ Another group of layered mixed-anion oxides, Ca_4_Sb_2_O and Ca_4_Bi_2_O, were predicted to have average p-type *ZT* values of 1.58 and 2.14 at 1000 K, respectively.^31^ Moreover, LaCuSeO, a structural analogue of BiCuSeO, was found to exhibit an average n-type *ZT* of 1.46, but its p-type performance is poor.^32^ Bi_2_O_2_Se was predicted to have a maximum p-type average *ZT* of 2.62 at 900 K.^33^ Recently, to overcome the poor p-type performance of LaCuSeO, Tang *et al.*^34^ proposed a novel van der Waals intercalated compound, La_2_Bi_4_Cu_2_O_6_Se_4_, by alternately stacking LaCuSeO and Bi_2_O_2_Se units along the *c*-axis in a 1 : 2 molar ratio using a high-entropy strategy. Computational results predict that this material could achieve a maximum p-type average *ZT* of 2.3 at 700 K. Notably, all these materials exhibit low lattice thermal conductivities below 1 W m^−1^ K^−1^ at temperatures corresponding to their optimal calculated *ZT* values. These theoretical predictions suggest that these layered mixed-anion oxides warrant experimental validation and further exploration.

Recently, layered mixed-anion oxides Bi_2_MO_4_Cl (M = Y, La, and Bi) with a triple-fluorite structure have attracted research attention for their photocatalytic activity, as they show potential as photocatalysts due to their suitable band potentials.^35^ However, the TE potential of these materials has never been explored. Considering the heavy elements (Bi, Y, and La), the layered structure (Cl^−^ layers and [Bi_2_MO_4_]^+^ layers), and the mixed-anion strategy (O^2−^ and Cl^−^) contributing to low lattice thermal conductivity, we predicted the electronic and thermal transport properties of Bi_2_MO_4_Cl (M = Y, La, and Bi) using density functional theory (DFT) calculations. Our results indicate that Bi_2_MO_4_Cl (M = Y, La, and Bi) possesses average electronic transport properties, but because of the heavy elements, weak ionic bonding, high Grüneisen parameters, and low structural symmetry, all three compounds exhibit intrinsically low lattice thermal conductivities, which further determines the high *ZT* values at 1000 K for Bi_2_LaO_4_Cl (1.74) and Bi_3_O_4_Cl (2.20). In addition, the weak ionic bonding formed between Cl^−^ and Bi^3+^ inhibits out-of-plane heat transfer, enabling the out-of-plane *ZT* of Bi_2_LaO_4_Cl to exceed 4. These results indicate that Bi_2_MO_4_Cl (M = Y, La, and Bi) compounds are competitive for TE applications and demonstrate the unique advantages of layered mixed-anion compounds in the design of high-performance TE materials.

## Computational methods

The DFT calculations in this study were implemented in the Vienna *Ab initio* Simulation Package (VASP).^36^ The interaction between core and valence electrons was accounted for using the projector augmented-wave (PAW) pseudopotential method.^37,38^ The energy cutoffs and the *Γ*-centred *k*-point meshes of the valence wavefunctions chosen to simulate the unit cell are shown in Table 1. The primitive unit cell of Bi_2_MO_4_Cl (M = Y or La) is the same as the conventional unit cell in each case, and hence the same *k*-point mesh was used. These parameters were selected to converge the total energy to within 1 meV per atom. The convergence tests for the energy cutoff and *k*-point mesh are presented in Section 1 of the SI.

**Table 1:** Converged energy cutoffs and *k*-point meshes used in this study, interpolation meshes used in calculating the electronic transport properties using AMSET, supercell meshes used in calculating the second- and third-order FCs using Phonopy and Phono3py, the number of displacements to be evaluated for calculating the third-order FCs, and *q*-point sampling meshes used in calculating the lattice thermal conductivity using Phono3py for Bi_2_MO_4_Cl (M = Y, La, and Bi)

Compound	Bi_2_YO_4_Cl	Bi_2_LaO_4_Cl	Bi_3_O_4_Cl (conventional cell)	Bi_3_O_4_Cl (primitive cell)
Energy cutoff	550 eV	550 eV	700 eV	700 eV
*K*-point mesh	6 × 6 × 3	2 × 2 × 4	1 × 4 × 4	4 × 4 × 3
Interpolation mesh	89 × 89 × 39	13 × 11 × 21	—	37 × 37 × 35
2nd supercell	5 × 5 × 2	2 × 2 × 3	1 × 4 × 4	—
3rd supercell	4 × 4 × 2	2 × 1 × 2	1 × 2 × 2	—
3rd calculations	5195	36 912	18 457	—
*Q*-point mesh	15 × 15 × 15	10 × 10 × 10	10 × 10 × 10	—

The primitive unit cells of Bi_2_MO_4_Cl (M = Y, La, and Bi) were fully relaxed using the hybrid Heyd–Scuseria–Ernzerhof (HSE06) functional^39,40^ until the maximum force on any atom was less than 0.0005 eV Å^−1^. The optimised structures were used for the calculation of electronic transport properties. To obtain the lattice thermal conductivities of Bi_2_MO_4_Cl (M = Y, La, and Bi), the Perdew–Burke–Ernzerhof functional revised for solids (PBEsol)^41^ of the Generalised Gradient Approximation (GGA) was used to sufficiently relax the conventional cells of Bi_2_YO_4_Cl and Bi_2_LaO_4_Cl until the maximum force on any atom is smaller than 0.0001 eV Å^−1^. In these processes of structural optimisation, a 30% higher plane-wave energy cut-off was used to counteract Pulay stresses.^42^ As the failure of the PBEsol functional to optimise the structure of Bi_3_O_4_Cl resulted in the presence of significant imaginary frequencies in its phonon dispersion (Fig. S3(b)), we used the GGA Perdew–Burke–Ernzerhof (PBE) functional,^43^ the meta-GGA regularised revised Strongly Constrained and Appropriately Normed (r^2^SCAN) functional^44^ and the hybrid HSE06 functional to optimise the Bi_3_O_4_Cl structure and calculate the phonon dispersion to determine which functional should be used for the evaluation of the second- and third-order force constants (FCs). By comparing the optimised lattice parameters with the experimental data, we chose the r^2^SCAN functional to evaluate the FCs, taking into account both the accuracy and the computational expense. More details on this section can be found in Section 2 of the SI. The electronic band structures and densities of states (DoS) were calculated using the HSE06 functional incorporating the effects of spin–orbit coupling (SOC) and analysed using the Sumo^45^ software package. We employed the Bradley–Cracknell formalism^46^ to determine the high-symmetry paths within the first Brillouin zone for the band structure representation.

To obtain the Seebeck coefficient, conductivity, and electronic thermal conductivity, AMSET^47^ was used to solve the electronic Boltzmann transport equation (BTE). Given the limitations of the constant relaxation-time approximation (CRTA) when solving the transport equation, AMSET improves computational accuracy by using the momentum relaxation-time approximation (MRTA) instead of CRTA.^47,48^ AMSET can evaluate the scattering caused by the acoustic deformation potential (ADP), ionised impurities (IMP), piezoelectric interactions (PIE), and polar optical phonons (POP). ADP, IMP, and POP scattering were considered in this article and the characteristic scattering *τ*_e_ was calculated according to Matthiessen's rule:2
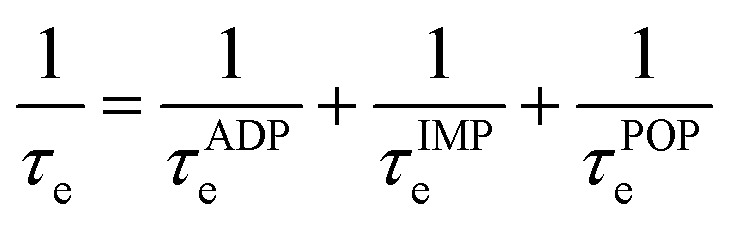


PIE scattering was not included because the calculated piezoelectric coefficient matrices for Bi_2_MO_4_Cl (M = Y, La, and Bi) were all zero, thus Bi_2_MO_4_Cl (M = Y, La, and Bi) was not piezoelectric. The parameter settings for AMSET calculations and the interpolation mesh tests for electronic transport properties are provided in Section 3 and Section 4 of the SI. Table 1 presents the interpolation meshes for Bi_2_YO_4_Cl, Bi_2_LaO_4_Cl and Bi_3_O_4_Cl used in this paper.

The second-order and third-order FCs required for calculating the lattice thermal conductivity were obtained using the finite displacement method in the Phonopy^49^ and Phono3py^50^ software packages, respectively. During the construction of the supercells, default displacements of 0.01 Å for Phonopy and 0.03 Å for Phono3py were used. Each displacement was evaluated using the PBEsol functional for Bi_2_YO_4_Cl and Bi_2_LaO_4_Cl, and the r^2^SCAN functional for Bi_3_O_4_Cl. The phonon dispersions of Bi_2_MO_4_Cl (M = Y, La, and Bi) were calculated for different supercell meshes (Fig. S5), and it was determined that the phonon dispersions converge at supercell meshes of 4 × 4 × 2, 2 × 1 × 2 and 1 × 2 × 2 for Bi_2_YO_4_Cl, Bi_2_LaO_4_Cl and Bi_3_O_4_Cl, respectively. The supercell meshes used in the evaluation of the second- and third-order FCs and the number of displacements used for calculating the third-order FCs have been listed in Table 1. Non-analytical corrections (NAC) were included to account for long-range interactions. Under the single-mode relaxation-time approximation (SM-RTA), the lattice thermal conductivity was calculated by solving the linearised BTE3
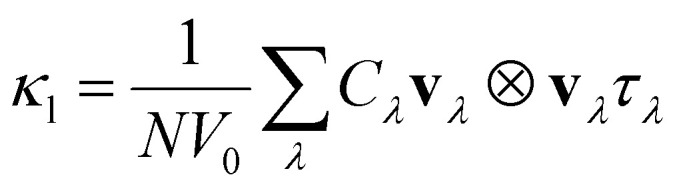
where *N* is the number of unit cells in the crystal, *V*_0_ is the volume of the unit cell, and **v**_*λ*_ and *τ*_*λ*_ are the group velocity and the phonon lifetime of the phonon mode *λ*, respectively. *C*_*λ*_ is the mode-dependent heat capacity. The *q*-point sampling tests of the lattice thermal conductivity of Bi_2_MO_4_Cl (M = Y, La, and Bi) are shown in Fig. S6 and the converged *q*-point sampling meshes for Bi_2_YO_4_Cl, Bi_2_LaO_4_Cl and Bi_3_O_4_Cl used in this paper are given in Table 1.

## Results and discussion

### Crystal structure

**Fig. 1:**
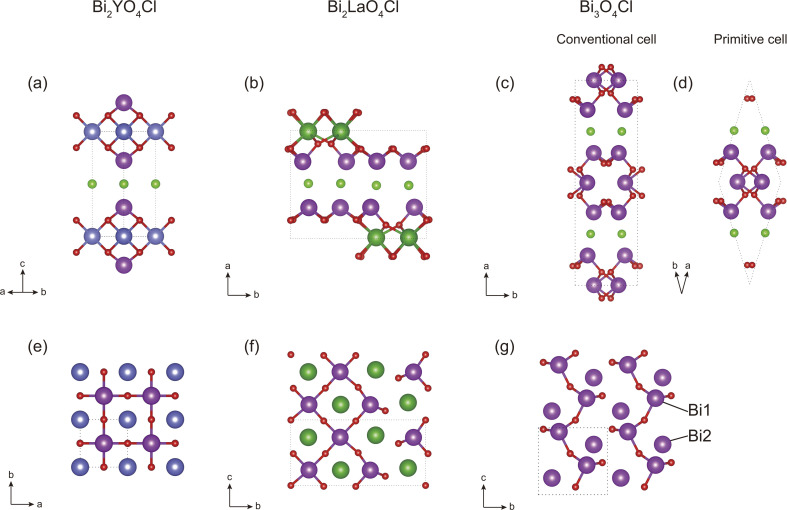
(a)–(d) are side views of (a) Bi_2_YO_4_Cl, (b) Bi_2_LaO_4_Cl, and (c) and (d) Bi_3_O_4_Cl. (e)–(g) are top views of the [Bi_2_YO_4_]^+^ layer of (e) Bi_2_YO_4_Cl, (f) Bi_2_LaO_4_Cl, and (g) Bi_3_O_4_Cl, with all M–O bonds and one BiO_2_ sublayer omitted for clarity. A unit cell is shown within the dotted line. The atoms are coloured as follows: Bi-purple, Y-blue, La-deep green, O-red and Cl-green. In order to distinguish between the Bi and M atoms of Bi_3_O_4_Cl, the three-coordinated Bi atom is labelled as Bi1, and the four-coordinated M (Bi) atom as Bi2. The images were generated using the VESTA software.^52^

The structures of Bi_2_MO_4_Cl (M = Y, La, and Bi) exhibit layered characteristics, consisting of alternating stacks of Cl^−^ layers and [Bi_2_MO_4_]^+^ layers. Bi_2_YO_4_Cl crystallises in the space group *P*4/*mmm* (no. 123), and exhibits high structural symmetry (Fig. 1). In the [Bi_2_YO_4_]^+^ layer, the outermost Bi atom is coordinated by four equidistant O atoms, and the Y atom forms an eight-coordination with eight equidistant O atoms and connects a double-layer two-dimensional grid composed of [BiO_4_] in the shape of a square pyramid. For Bi_2_LaO_4_Cl, the powder X-ray diffraction data with the selected area electron diffraction patterns of Nakada *et al.*^35^ clearly showed monoclinic distortion and found that the *P*2_1_/*a* (no. 14) space group was the most probable, but Milne *et al.*^51^ used the ideal *P*4/*mmm* structure. We chose the *P*2_1_/*a* structure of Bi_2_LaO_4_Cl because we are mainly interested in the TE performance from 100 K to 1000 K and the free energy of the *P*2_1_/*a* structure is lower in this temperature range (Fig. S7). In practical calculations, we used the standard form *P*2_1_/*c* of space group *P*2_1_/*a*. The structure of Bi_2_LaO_4_Cl is similar to that of Bi_2_YO_4_Cl, with the difference that in the outermost region of the [Bi_2_LaO_4_]^+^ layer, half of the Bi atoms become three-coordinated, while the other half remain four-coordinated. Bi_3_O_4_Cl crystallises in the space group *I*2/*a* (no. 15), and again in practical calculations we used the standard form *C*2/*c* of this numbered space group. In the [Bi_2_BiO_4_]^+^ layer of Bi_3_O_4_Cl, the outermost Bi atoms (Bi1) all become three-coordinated, and the M atoms (Bi2) remain in the middle of the [Bi_2_BiO_4_]^+^ layer.

The experimental and calculated lattice parameters of Bi_2_MO_4_Cl (M = Y, La, and Bi) are given in Table 2, and the differences between the calculated and experimental values are within reasonable limits.

**Table 2:** Calculated lattice parameters of Bi_2_MO_4_Cl (M = Y, La, and Bi). The percentage differences from experimental parameters are given in parentheses. The experimental lattice parameters for the *P*2_1_/*c* (*C*2/*c*) structure of Bi_2_LaO_4_Cl (Bi_3_O_4_Cl) were converted from the experimental lattice parameters for the *P*2_1_/*a* (*I*2/*a*) structure

Compound		*a* (Å)	*b* (Å)	*c* (Å)
Bi_2_YO_4_Cl	Nakada *et al.* (exp.^35^)	3.85	3.85	8.89
	PBEsol	3.84 (−0.26%)	3.84 (−0.26%)	8.83 (−0.67%)
	HSE06	3.85 (0.00%)	3.85 (0.00%)	8.99 (1.12%)
Bi_2_LaO_4_Cl	Nakada *et al.* (exp.^35^)	9.02	11.55	5.59
	PBEsol	8.98 (−0.44%)	11.46 (−0.78%)	5.60 (0.18%)
	HSE06	9.14 (1.33%)	11.55 (0.00%)	5.61 (0.36%)
Bi_3_O_4_Cl	Nakada *et al.* (exp.^35^)	19.28	5.65	5.69
	r^2^SCAN	19.45 (0.88%)	5.70 (0.88%)	5.73 (0.70%)
	HSE06	19.54 (1.35%)	5.72 (1.24%)	5.76 (1.23%)

### Electronic structure and phonon dispersion

**Fig. 2:**
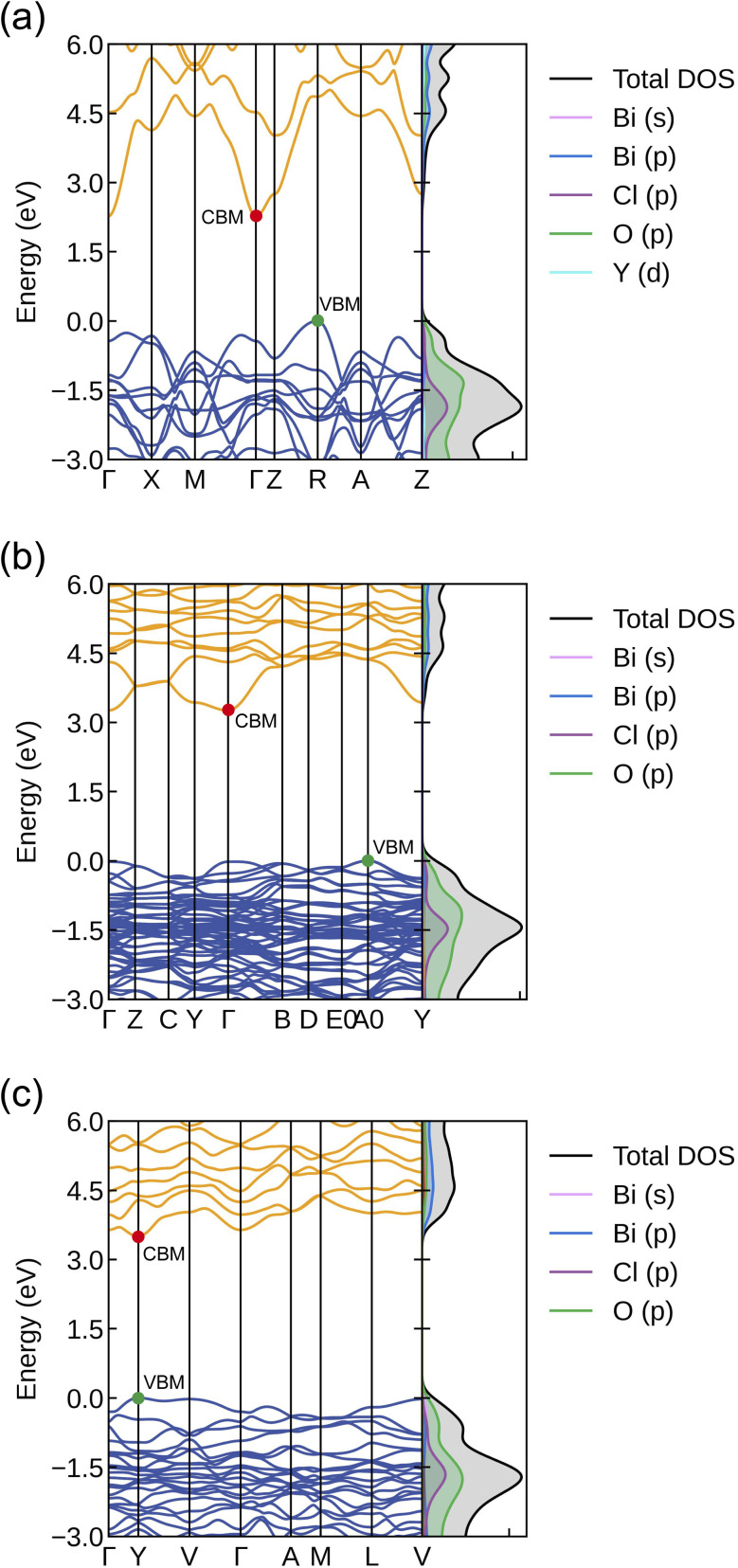
Electronic band structures and DoS of (a) Bi_2_YO_4_Cl, (b) Bi_2_LaO_4_Cl, and (c) Bi_3_O_4_Cl calculated by using the HSE06 functional with SOC, plotted along the Bradley–Cracknell-derived *k*-point path.^46^ The conduction and valence bands are coloured by orange and blue. These figures were generated using Sumo.^45^

Fig. 2 shows the electronic band structures and DoS of Bi_2_MO_4_Cl (M = Y, La, and Bi) calculated using the HSE06 functional with SOC. Bi_2_YO_4_Cl (Bi_2_LaO_4_Cl) is an indirect band gap semiconductor with the valence band maximum (VBM) located at the symmetry point R (A_0_), the conduction band minimum (CBM) located at the symmetry point Γ (Γ). The direct band gap is 2.70 eV (3.28 eV) and the indirect band gap is 2.27 eV (3.26 eV). Bi_3_O_4_Cl is a direct band gap semiconductor, with the VBM and CBM located at the symmetry point Y and a band gap of 3.50 eV. Both Bi_2_LaO_4_Cl and Bi_3_O_4_Cl have flatter bands, indicating smaller carrier effective masses and thus higher carrier mobilities. As shown in Fig. 2, The VBM of Bi_2_MO_4_Cl (M = Y, La, and Bi) is dominated by O 2p, while the CBM is primarily contributed by Bi 6p.

**Fig. 3:**
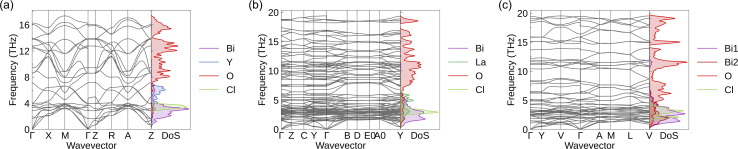
Phonon dispersions and atom-projected phonon DoS of (a) Bi_2_YO_4_Cl, (b) Bi_2_LaO_4_Cl, and (c) Bi_3_O_4_Cl with NAC applied. The phonon dispersions were plotted using ThermoParser,^54^ and the high-symmetry paths were constructed using the Bradley–Cracknell formalism.^46^

Fig. 3 shows the phonon dispersions and atom-projected phonon DoS of Bi_2_MO_4_Cl (M = Y, La, and Bi). None of the phonon dispersions of the three compounds have imaginary frequencies, so they all satisfy dynamical stability. As the mass of the M atom increases, the phonon branches tend to be flatter. A flat phonon branch implies low phonon group velocity, since the phonon group velocity is defined as the derivative of the phonon frequency with respect to the wave vector. Moreover, the frequency ranges of the acoustic modes are compressed. The acoustic-mode boundary frequency of Bi_2_YO_4_Cl is ∼4 THz, while those of Bi_2_LaO_4_Cl and Bi_3_O_4_Cl are both close to 2 THz. The latter two materials have acoustic-mode boundary frequencies similar to those of the known low-thermal-conductivity materials BiCuSeO and SnSe, which also exhibit layered structural characteristics.^27,32,53^ This similarity suggests that Bi_2_LaO_4_Cl and Bi_3_O_4_Cl may also possess low lattice thermal conductivity. The high-frequency phonon modes in the three compounds are dominated by the vibrations of O atoms, and the low-frequency phonon modes consist mainly of the vibrations of Cl, M (M = Y, La, and Bi) and Bi atoms. Interestingly, the lighter Cl atoms contribute more to the lower-frequency (3–4 THz) phonons than the heavier Y and La atoms. This phenomenon can be attributed to the softening of the Cl-dominated optical phonon modes caused by the weak Bi–Cl bonds, which will be discussed in detail later in this section. In addition, some of the optical modes are located in the low-frequency region of the dispersion, which would provide more channels for phonon scattering.

### Transport and TE properties

In this section, we analyse the transport and TE properties of Bi_2_MO_4_Cl (M = Y, La, and Bi). The electronic transport properties of Bi_2_MO_4_Cl (M = Y, La, and Bi) were calculated using the AMSET package, while the lattice thermal conductivity was determined using Phono3py. The combination of these results was used to predict the *ZT* values of Bi_2_MO_4_Cl (M = Y, La, and Bi). The temperature range of the calculations was chosen to be from 100 K to 1000 K because Bi_2_YO_4_Cl and Bi_2_LaO_4_Cl were synthesised in air at 1073 K, and for Bi_3_O_4_Cl at 973 K.^35^ The p-type performance of Bi_2_MO_4_Cl (M = Y, La, and Bi) is discussed in detail, as it is significantly better than the n-type performance. Section 8 of the SI presents the n-type electronic transport properties and *ZT* values of Bi_2_MO_4_Cl (M = Y, La, and Bi).

**Fig. 4:**
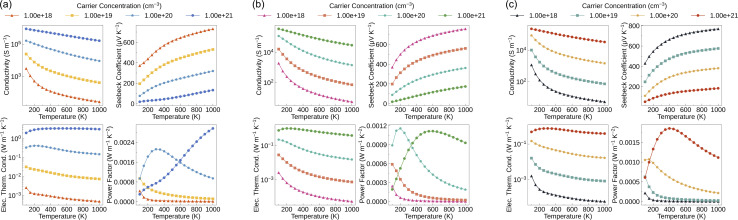
Calculated p-type electronic transport properties as a function of temperature for (a) Bi_2_YO_4_Cl, (b) Bi_2_LaO_4_Cl and (c) Bi_3_O_4_Cl with four different carrier concentrations.

Fig. 4 shows the p-type electronic transport properties of Bi_2_MO_4_Cl (M = Y, La, and Bi). The conductivity of Bi_2_MO_4_Cl (M = Y, La, and Bi) consistently decreases with increasing temperature and always increases with increasing carrier concentration. These behaviours can be explained by the equation for conductivity4
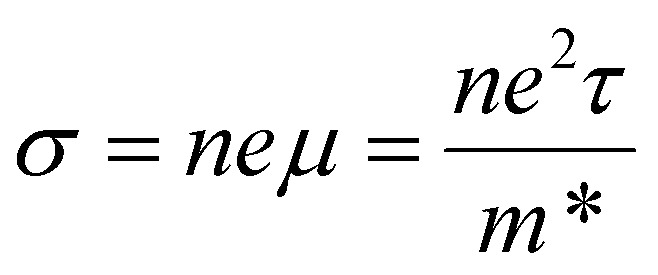
where *n* is the carrier concentration, *e* is the charge of an electron, *μ* is the carrier mobility, *τ* is the carrier lifetime, and m* is the carrier effective mass. At the same carrier concentration, the total scattering rates increase with increasing temperature (Fig. S10(a), (d), and (g)), which leads to the decrease of the carrier lifetime and hence the conductivity. Conversely, increasing the carrier concentration enhances the conductivity not only because of the direct proportionality but also due to the progressively decreasing total scattering rates (Fig. S10(b), (e), and (h)). In addition, at low carrier concentrations, conductivity exhibits a stronger temperature dependence because the strongly temperature-dependent POP scattering rates decrease, while the weakly temperature-dependent IMP scattering rates increase with increasing carrier concentration. The range of conductivity narrows as the M (Y → La → Bi) element changes. Specifically, the conductivity ranges from 27.3 to 9.00 × 10^5^ S m^−1^ for Bi_2_YO_4_Cl, from 5.47 to 3.54 × 10^5^ S m^−1^ for Bi_2_LaO_4_Cl, and from 5.35 to 2.27 × 10^5^ S m^−1^ for Bi_3_O_4_Cl, at carrier concentrations ranging from 10^18^ to 10^21^ cm^−3^. Compared to the calculated conductivity of BiCuOSe, Bi_2_MO_4_Cl (M = Y, La, and Bi) exhibit higher conductivity at 300 K, 600 K, and 900 K for a carrier concentration of 1 × 10^20^ cm^−3^.^32^ This carrier concentration was chosen because the maximum *ZT* values were obtained at this magnitude for Bi_2_MO_4_Cl (M = Y, La, and Bi) and BiCuOSe. However, at the same carrier concentration and at 1000 K, the calculated electrical conductivity of the state-of-the-art TE material SnSe is approximately 6.8 × 10^4^ S m^−1^,^53^ which is an order of magnitude higher than that of the Bi_2_MO_4_Cl series, with values of 9.24 × 10^3^ S m^−1^ for Bi_2_YO_4_Cl, 1.43 × 10^3^ S m^−1^ for Bi_2_LaO_4_Cl, and 1.48 × 10^3^ S m^−1^ for Bi_3_O_4_Cl.

The Seebeck coefficient measures the ability of a material to generate an electric potential in response to a temperature difference. Low carrier concentration and large carrier effective mass provide high Seebeck coefficient:5
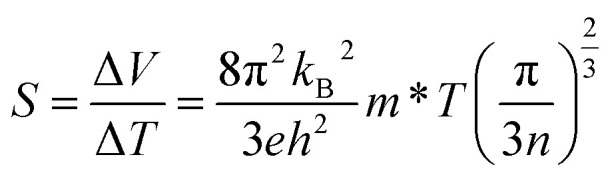
where *V* is the voltage, *k*_B_ is the Boltzmann constant, and *h* is Planck's constant, thus the Seebeck coefficient is inversely proportional to the conductivity. At a temperature of 900 K and a carrier concentration of 1 × 10^20^ cm^−3^, the Seebeck coefficient of BiCuOSe (∼380 μV K^−1^) is comparable to that of Bi_3_O_4_Cl (373 μV K^−1^) and slightly higher than that of Bi_2_LaO_4_Cl (347 μV K^−1^) and Bi_2_YO_4_Cl (301 μV K^−1^). However, a high power factor requires a balance between the conductivity and the Seebeck coefficient. The p-type power factors of Bi_2_MO_4_Cl (M = Y, La, and Bi) span the ranges from 14.6 to 2960, 3.16 to 1160, and 3.16 to 1850 μW m^−1^ K^−2^ at carrier concentrations of 10^18^ to 10^21^ cm^−3^, respectively. In contrast, the maximum power factor of BiCuOSe is only around 770 μW m^−1^ K^−2^ at carrier concentrations ranging from 10^19^ to 10^22^ cm^−3^.^32^ That is why the intrinsic *ZT* (0.75) of BiCuOSe is modest despite its low thermal conductivity, and its TE performance is limited by the low conductivity.^32,55^ The power factors of Bi_2_LaO_4_Cl and Bi_3_O_4_Cl are also relatively low, much smaller than the maximum power factor (∼4000 μW m^−1^ K^−2^) obtained in the calculations for SnSe,^53^ and there is also a significant gap between them and some newly reported high-performance p-type TE materials.^56,57^ These results indicate that Bi_2_LaO_4_Cl and Bi_3_O_4_Cl cannot be regarded as compounds with excellent electronic transport properties.

The total thermal conductivity is obtained by summing the electronic thermal conductivity and the lattice thermal conductivity. The Wiedemann–Franz law links the conductivity to the electronic thermal conductivity

**Fig. 5:**
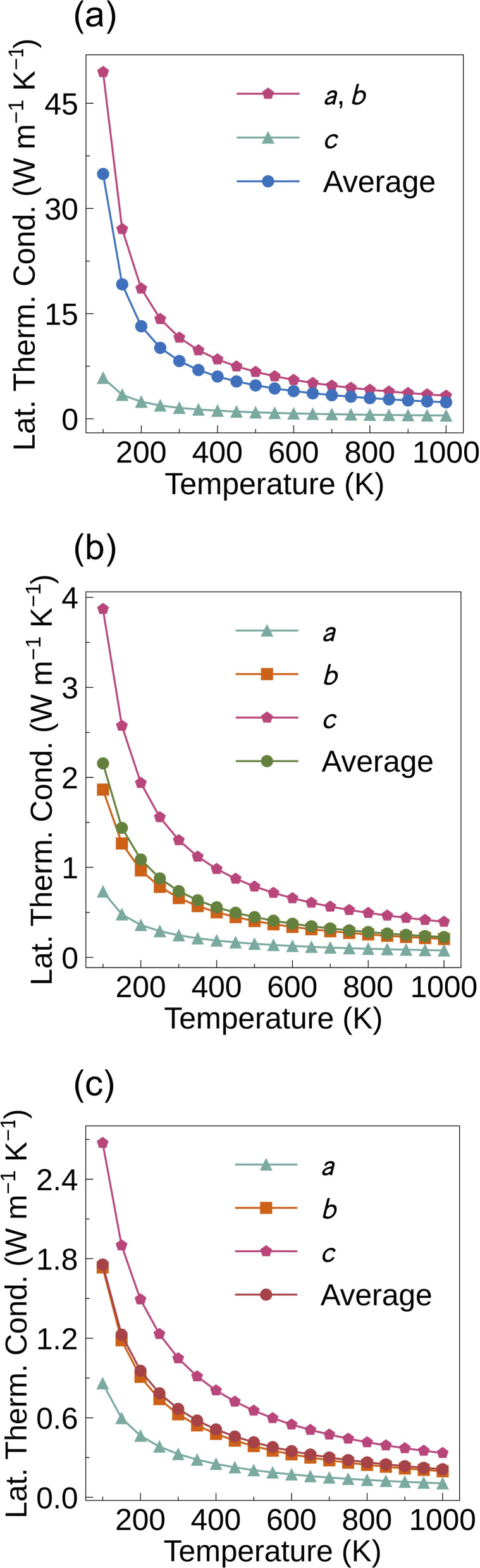
Calculated lattice thermal conductivities of (a) Bi_2_YO_4_Cl, (b) Bi_2_LaO_4_Cl and (c) Bi_3_O_4_Cl in the *a*, *b* and *c* directions as a function of temperature.

6 *κ*_e_ = *LσT*

where *L* is the Lorentz number. As the carrier concentration varies, the trend of the electronic thermal conductivity is similar to that of the conductivity. The lattice thermal conductivities of Bi_2_MO_4_Cl (M = Y, La, and Bi) (Fig. 5) show that the low-symmetry Bi_2_LaO_4_Cl and Bi_3_O_4_Cl have ultra-low lattice thermal conductivities, which is favourable for achieving high *ZT* values. At 1000 K, the average lattice thermal conductivities of Bi_2_YO_4_Cl, Bi_2_LaO_4_Cl and Bi_3_O_4_Cl are 2.39, 0.23 and 0.21 W m^−1^ K^−1^ (Table 3), respectively. Compared to several p-type TE materials with low calculated lattice thermal conductivity (BiCuOSe (0.36 W m^−1^ K^−1^), SnSe (0.47 W m^−1^ K^−1^), Ca_4_Sb_2_O (0.60 W m^−1^ K^−1^), Ca_4_Bi_2_O (0.33 W m^−1^ K^−1^), Bi_2_O_2_Se (0.87 W m^−1^ K^−1^), La_2_Bi_4_Cu_2_O_6_Se_4_ (0.78 W m^−1^ K^−1^), Ca_6_NFSn_2_ (0.88 W m^−1^ K^−1^), Sr_6_NFSn_2_ (0.25 W m^−1^ K^−1^), Sb_2_Si_2_Te_6_ (0.35 W m^−1^ K^−1^) and Sc_2_Si_2_Te_6_ (0.97 W m^−1^ K^−1^)), Bi_2_LaO_4_Cl and Bi_3_O_4_Cl have even lower average lattice thermal conductivities.^31–34,53,56,57^ It is worth noting that in the system we studied, the lowest lattice thermal conductivity is always observed in the direction perpendicular to the layers (the *c* direction for Bi_2_YO_4_Cl and the *a* direction for Bi_2_LaO_4_Cl and Bi_3_O_4_Cl) compared to other directions. We will present the reasons for this in the next section. When calculating the lattice thermal conductivity under SM-RTA, only the three-phonon scattering mechanism is considered, while other scattering processes such as higher-order phonon scattering and phonon-defect scattering are ignored. Additionally, SM-RTA fails to account for the collective phonon excitation effects included in the full solution of the linearised BTE, leading to an underestimation of the lattice thermal conductivity.^58,59^ However, the neglect of collective phonon excitation partially compensates for the omission of other scattering mechanisms, resulting in computational results that generally agree well with experimental values.^25,26,32,60^

**Table 3:** Predicted p-type maximum *ZT* values at 1000 K for Bi_2_MO_4_Cl (M = Y, La, and Bi) with the corresponding carrier concentration (*n*), lattice thermal conductivity (*κ*_l_), power factor (PF) and electronic thermal conductivity (*κ*_e_)

Compound	Direction	*n* (cm^−3^)	max *ZT*	*κ*_l_ (W m^−1^ K^−1^)	PF (μW m^−1^ K^−2^)	*κ*_e_ (W m^−1^ K^−1^)
Bi_2_YO_4_Cl	*a*, *b*	4.64 × 10^20^	0.60	3.30	2840	1.40
	*c*	2.15 × 10^20^	1.78	0.46	1300	0.27
	Average	3.16 × 10^20^	0.69	2.35	2110	0.71
Bi_2_LaO_4_Cl	*a*	3.16 × 10^20^	4.13	0.077	586	0.065
	*b*	6.81 × 10^20^	0.97	0.20	320	0.13
	*c*	6.81 × 10^20^	1.47	0.40	1160	0.39
	Average	4.64 × 10^20^	1.74	0.23	642	0.14
Bi_3_O_4_Cl	*a*	4.64 × 10^20^	2.52	0.11	555	0.11
	*b*	6.81 × 10^20^	1.97	0.20	730	0.17
	*c*	4.64 × 10^20^	2.20	0.33	1190	0.21
	Average	4.64 × 10^20^	2.20	0.21	770	0.14

**Fig. 6:**
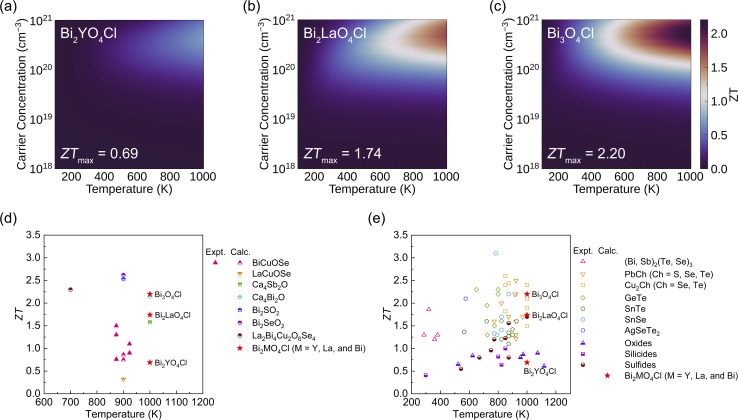
Predicted p-type *ZT* of (a) Bi_2_YO_4_Cl, (b) Bi_2_LaO_4_Cl and (c) Bi_3_O_4_Cl. This analysis was conducted using ThermoParser.^54^ Comparison of p-type *ZT* values with (d) layered mixed-anion oxides BiCuOSe,^25–27,29,32,60,114^ LaCuOSe,^32^ Ca_4_Sb_2_O and Ca_4_Bi_2_O,^31^ Bi_2_SO_2_ and Bi_2_SeO_2_,^33^ and La_2_Bi_4_Cu_2_O_6_Se_4_ (ref. 34) and (e) telluride semiconductors and their chalcogenide analogues that have been extensively studied (Bi, Sb)_2_(Te, Se)_3_,^61–64^ PbCh (Ch = S, Se, Te),^20,65–73^ Cu_2_Ch (Ch = Se, Te),^74–82^ SnSe,^19,115–119^ SnTe,^83–90^ GeTe,^91–98^ AgSeTe_2_,^99,100^ as well as environmentally friendly oxides,^5,15–17^ silicides,^101–104^ and sulfides.^105–112^

Finally, *ZT* was calculated using ThermoParser^54^ in conjunction with the calculated lattice thermal conductivity and electronic transport properties (Fig. 6(a–c) and Table 3). Due to the large average lattice thermal conductivity, the maximum average *ZT* of Bi_2_YO_4_Cl is only 0.69, but the out-of-plane *ZT* (1.78) is very high. The maximum average *ZT* values of Bi_2_LaO_4_Cl and Bi_3_O_4_Cl are 1.74 and 2.20, respectively, indicating good TE conversion ability, and the out-of-plane *ZT* of Bi_2_LaO_4_Cl is over 4. Furthermore, we compared the average maximum *ZT* values of Bi_2_MO_4_Cl (M = Y, La, and Bi) at 1000 K with those of layered mixed-anion oxides, telluride semiconductors and their chalcogenide analogues, as well as environmentally friendly oxides, silicides, and sulfides (Fig. 6(d) and (e)). Currently, research on layered mixed-anion oxide TE materials remains limited; hence, the reported high *ZT* values (Ca_4_Bi_2_O (2.14), Bi_2_SO_2_ (2.53), Bi_2_SeO_2_ (2.62), and La_2_Bi_4_Cu_2_O_6_Se_4_ (2.3))^31,33,34^ are all derived from computational studies. The optimal *ZT* of Bi_3_O_4_Cl is comparable to those of these high-performance materials. Compared with the *ZT* values of telluride semiconductors and their chalcogenide analogues, the optimal *ZT* of Bi_3_O_4_Cl is lower than that of polycrystalline SnSe (3.1) with tin oxide removed,^19^ but it is similar to the *ZT* values of other telluride semiconductor materials and their chalcogenide analogues,^20,61–100^ and significantly superior to those of environmentally friendly oxides, silicides, and sulfides.^5,15–17,101–112^ Moreover, Bi_2_MO_4_Cl (M = Y, La, and Bi) contain no highly toxic elements. Oxygen and chlorine are earth-abundant, while yttrium and lanthanum are relatively resource-rich. Bismuth offers a stable supply and moderate cost. Therefore, this system demonstrates environmental friendliness and resource sustainability. The TE performance of Bi_2_LaO_4_Cl is also at a high level in environmentally friendly materials. It should be noted that the optimal *ZT* values of Bi_2_MO_4_Cl (M = Y, La, and Bi) depend on high carrier concentrations (>10^20^ cm^−3^). As a result, a comprehensive understanding of defect/doping chemistry and a rational design of doping strategies are essential to obtain the predicted TE performance in practice.^113^ Although it is known from Fig. 4 and Table 3 that Bi_2_LaO_4_Cl and Bi_3_O_4_Cl are not materials with excellent electronic transport properties, their ultra-low lattice thermal conductivities result in high average *ZT* values. Therefore, the origin of the low lattice thermal conductivity of Bi_2_MO_4_Cl (M = Y, La, and Bi) will be discussed in the next section.

### Origin of low lattice thermal conductivity

The lattice thermal conductivity of a system is primarily determined by phonon group velocity and phonon lifetime, according to eqn (3). The heat capacity is generally not considered a major factor influencing the lattice thermal conductivity, since it is only weakly dependent on the phonon frequency.

**Fig. 7:**
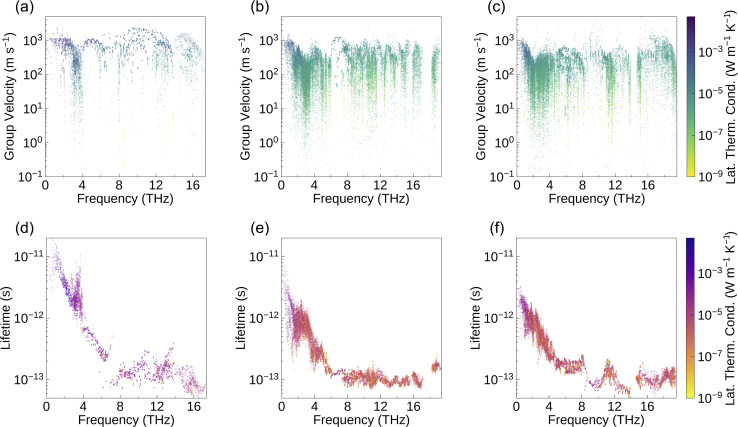
(a)–(c) Phonon group velocities and (d)–(f) phonon lifetimes (the latter calculated at 1000 K) for Bi_2_YO_4_Cl, Bi_2_LaO_4_Cl and Bi_3_O_4_Cl. The lattice thermal conductivity at 1000 K is projected on the colour axis, with yellow denoting low lattice thermal conductivity and deep blue denoting high lattice thermal conductivity.

The phonon group velocity of Bi_2_MO_4_Cl (M = Y, La, and Bi) is in the range of 1 × 10^−1^ to 5 × 10^3^ m s^−1^ (Fig. 7(a)–(c)), with average values of 680 m s^−1^, 319 m s^−1^, and 367 m s^−1^, respectively. The phonon group velocity is related to atomic mass and bond strength. Heavy elements and weak bonds contribute to the softness of phonon modes, thereby reducing phonon group velocities. Since the three compounds contain heavy elements such as Y, La, and Bi, their phonon group velocities are generally lower. In addition, we calculated the average negative Integrated Crystal Orbital Hamiltonian Population (–ICOHP) for each chemical bond in Bi_2_MO_4_Cl (M = Y, La, and Bi) using Lobster,^120^ as shown in Table 4. The COHP describes the contribution of a particular bond to the stability of the whole system, while the ICOHP represents the result of integrating the COHP over a range of energies and is commonly used to quantify the strength of interatomic bonds. We find that the Bi–Cl bond in Bi_2_MO_4_Cl (M = Y, La, and Bi) is characterised by weak ionic character, with a significantly lower average –ICOHP than those of the strongly covalent Bi–O and La–O bonds. The weak Bi–Cl bond slows down the group velocity of phonons. The –ICOHP results also explain the anomaly that we observe in the phonon dispersions (Fig. 3(a) and (b)): the atomic masses of Y and La are heavier than that of Cl, but the vibrational contribution of Cl atoms is more significant in the lower frequency range (3–4 THz). This is because despite the heavier atomic masses of Y and La, the weak bonding strength of the Bi–Cl bond reduces the vibrational frequency of Cl atoms, making the low-frequency phonons produced by their vibrations lower in energy.

**Table 4:** Average –ICOHP per bond for Bi_2_MO_4_Cl (M = Y, La, and Bi)

Compound	M–O (eV)	Bi–O (eV)	Bi–Cl (eV)
Bi_2_YO_4_Cl	2.47	3.01	0.425
Bi_2_LaO_4_Cl	2.66	3.44	0.385
Bi_3_O_4_Cl	2.86	4.36	0.361

However, the average group velocities of Bi_2_LaO_4_Cl and Bi_3_O_4_Cl are nearly half of that of Bi_2_YO_4_Cl. This difference partly arises from the lower average atomic mass of Bi_2_YO_4_Cl. Additionally, the significant difference in average group velocity is also related to symmetry breaking. The high symmetry of Bi_2_YO_4_Cl (Fig. 1(a) and (e)) results in low-frequency acoustic modes exhibiting dispersion relations closely approaching those of ideal elastic waves in the long-wavelength limit (Fig. 3(a)), thus yielding larger slopes, *i.e.*, higher phonon group velocities. In contrast, the acoustic modes of the low-symmetry Bi_2_LaO_4_Cl and Bi_3_O_4_Cl exhibit more pronounced bending in the long-wavelength limit (Fig. 3(b) and (c)), leading to significantly reduced average group velocities.

The phonon lifetime is affected by the number of phonon scattering channels and the intensity of phonon scattering. The Grüneisen parameter is often used to assess the phonon anharmonicity of a material. The larger the value, the stronger the phonon anharmonicity, which also implies more intense phonon–phonon scattering.^22,121^ The Grüneisen parameters for Bi_2_YO_4_Cl, Bi_2_LaO_4_Cl, and Bi_3_O_4_Cl are 2.03, 1.77, and 1.46, respectively, and these values are higher than or close to the Grüneisen parameter of the low-thermal-conductivity material BiCuOSe (1.5).^26^ The equation for calculating the Grüneisen parameter is provided in Section 10 of the SI. Since a high Grüneisen parameter is one of the most important reasons for the low thermal conductivity of BiCuOSe, the effect of scattering intensity on the phonon lifetime of Bi_2_MO_4_Cl (M = Y, La, and Bi) cannot be neglected. It is worth noting that weak bonding usually leads to strong phonon anharmonicity.^22,23^ Hence, based on the average –ICOHP data (Table 4), the weak Bi–Cl bonds make a significant contribution to the high Grüneisen parameters of Bi_2_MO_4_Cl (M = Y, La, and Bi). On the other hand, the heavy elements and weak bonding affect not only the phonon group velocity, but they make a part of the optical modes located in the low-frequency region of the phonon dispersion, providing more channels for phonon scattering, and that is why there is a substantial reduction of the phonon lifetime in the low-frequency region (Fig. 7(d)–(f)). Furthermore, the large differences in bond strength (Table 4) fully explain the presence of an extremely strong bonding heterogeneity in Bi_2_MO_4_Cl (M = Y, La, and Bi). The weak Bi–Cl bond lowers the vibrational frequency of the Cl atoms, enabling the associated optical modes to emerge in the low-frequency part of the phonon dispersion. This is a key advantage of mixed-anion compounds, as it enables the modulation of the vibrational modes of light elements to reduce lattice thermal conductivity.^24^ Therefore, bonding heterogeneity influences the phonon lifetime in terms of both phonon scattering intensity and the number of phonon scattering channels.

Comparing the three compounds, Bi_2_YO_4_Cl exhibits substantially longer phonon lifetimes in the low-frequency range compared to the other two compounds (Fig. 7(d)–(f)). This difference is closely associated with symmetry breaking: the lower symmetry of Bi_2_LaO_4_Cl and Bi_3_O_4_Cl leads to the lifting of degeneracy and phonon branch splitting, thereby increasing the number of available phonon scattering channels. The phonon scattering phase space can be regarded as the weighted density of scattering channels, serving as a measure of available scattering channels under energy and momentum conservation. Bi_2_YO_4_Cl exhibits a smaller three-phonon scattering phase space throughout the entire frequency range (Fig. S13). Together with the previous analysis on phonon group velocities, this explains why the lattice thermal conductivity of Bi_2_YO_4_Cl is significantly higher than that of Bi_2_LaO_4_Cl and Bi_3_O_4_Cl. Furthermore, as shown in Fig. 7, Bi_2_YO_4_Cl has more phonon modes that contribute significantly to lattice thermal conductivity compared to Bi_2_LaO_4_Cl and Bi_3_O_4_Cl, both in terms of phonon group velocities and phonon lifetimes.

Moreover, the compounds we studied always exhibit the lowest lattice thermal conductivity in the direction perpendicular to the layers (the *c* direction for Bi_2_YO_4_Cl and the *a* direction for Bi_2_LaO_4_Cl and Bi_3_O_4_Cl) compared with other directions, which is mainly attributed to the weak ionic bonding. Since this comparison focuses on the lattice thermal conductivity of the same material along different orientations, the effect of atomic mass can be neglected. The structures of Bi_2_MO_4_Cl (M = Y, La, and Bi) consist of alternating stacks of Cl^−^ and [Bi_2_MO_4_]^+^ layers. The [Bi_2_MO_4_]^+^ layers are dominated by covalent bonding with strong interatomic interactions, which facilitates efficient phonon transport within the layers. However, in the out-of-plane direction, heat must be transferred through the Cl^−^ and [Bi_2_MO_4_]^+^ layers in an environment of alternating covalent and ionic bonding. The weak ionic bonding hinders phonon heat transport across the layers, which leads to a lower out-of-plane lattice thermal conductivity of Bi_2_MO_4_Cl (M = Y, La, and Bi) than the in-plane value, while also causing the out-of-plane *ZT* of Bi_2_LaO_4_Cl to exceed 4.

## Conclusions

In conclusion, we predicted the high *ZT* values of Bi_2_MO_4_Cl (M = Y, La, and Bi) without highly toxic elements. We found a maximum average *ZT* of 2.20 for Bi_3_O_4_Cl and above 4 for Bi_2_LaO_4_Cl along the out-of-plane direction. Although the maximum average *ZT* of Bi_2_YO_4_Cl is relatively low, it still has a high out-of-plane *ZT* (1.78). The calculated results indicate that Bi_2_MO_4_Cl (M = Y, La, and Bi) are not materials with excellent electronic transport properties, which means that these high *ZT* values are mainly due to the ultra-low lattice thermal conductivity. It is shown that heavy elements, weak ionic bonding, and low structural symmetry result in low phonon group velocity and enhance phonon scattering, while high Grüneisen parameters imply strong phonon anharmonicity, which collectively determine the ultra-low lattice thermal conductivity. In addition, the weak ionic bonding hinders out-of-plane heat transport, making the out-of-plane lattice thermal conductivity of Bi_2_MO_4_Cl (M = Y, La, and Bi) lower than that in the in-plane direction. It should be emphasised that yielding the predicted maximum *ZT* values requires relatively high carrier concentrations (*n* > 10^20^ cm^−3^). Therefore, the actual doping level must be sufficient to sustain such high carrier concentrations in order to realise the optimal performance. Our research reveals new environmentally friendly TE material candidates in layered mixed-anion oxides, and also displays the great potential of the layered mixed-anion strategy in developing high-performance TE materials.

## Author contributions

Shipeng Bi: methodology, software, investigation, data curation, formal analysis, validation, visualisation, writing – original draft, and writing – review & editing. Christopher N. Savory: methodology. Alexander G. Squires: formal analysis. Dan Han: formal analysis, and writing – review & editing. Kieran B. Spooner: formal analysis. David O. Scanlon: conceptualisation, funding acquisition, resources, project administration, supervision, writing – review & editing.

## Conflicts of interest

The authors declare no competing interests.

## Supplementary Material

TA-013-D5TA05523G-s001

## Data Availability

Calculation data and parsed outputs are provided in an openly available online repository at https://zenodo.org/records/15836123. The supplementary information includes energy cutoff and *k*-point mesh convergence tests, phonon dispersions of Bi_3_O_4_Cl, AMSET settings, convergence tests of the interpolation mesh for electronic transport properties, phonon supercell mesh convergence tests, convergence tests of lattice thermal conductivity with respect to *q*-point sampling, *P*4/*mmm* and *P*2_1_/*c* structures of Bi_2_LaO_4_Cl, n-type electronic transport properties and *ZT* values of Bi_2_MO_4_Cl (M = Y, La, and Bi), scattering rates, the heat-capacity-weighted root-mean-square (RMS) Grüneisen parameter, and the three-phonon scattering phase space. See DOI: https://doi.org/10.1039/d5ta05523g.

## References

[cit1] Tomlinson B., Torrance A. W., Ripple W. J. (2024). J. Cleaner Prod..

[cit2] Firth A., Zhang B., Yang A. (2019). Appl. Energy.

[cit3] Slack G. A. , in CRC Handbook of Thermoelectrics, ed. D. M. Rowe, CRC Press, Boca Raton, 1st edn, 1995, ch. 34, pp. 407–440

[cit4] Franz R., Wiedemann G. (1853). Ann. Phys..

[cit5] He X. Y., Kimura S., Katase T., Tadano T., Matsuishi S., Minohara M., Hiramatsu H., Kumigashira H., Hosono H., Kamiya T. (2024). Adv. Sci..

[cit6] Tan G. J., Zhao L. D., Kanatzidis M. G. (2016). Chem. Rev..

[cit7] Wu C. L., Shi X. L., Wang L. J., Lyu W. Y., Yuan P., Cheng L. N., Chen Z. G., Yao X. D. (2024). ACS Nano.

[cit8] Koumoto K., Wang Y. F., Zhang R. Z., Kosuga A., Funahashi R. (2010). Annu. Rev. Mater. Res..

[cit9] Feng Y. N., Jiang X. D., Ghafari E., Kucukgok B., Zhang C. Y., Ferguson I., Lu N. (2018). Adv. Compos. Hybrid Mater..

[cit10] Wu T. J., Gao P. (2018). Materials.

[cit11] Ohtaki M., Araki K., Yamamoto K. (2009). J. Electron. Mater..

[cit12] Wang J., Zhang B. Y., Kang H. J., Li Y., Yaer X. B., Li J. F., Tan Q., Zhang S., Fan G. H., Liu C. Y., Miao L., Nan D., Wang T. M., Zhao L. D. (2017). Nano Energy.

[cit13] Li J. B., Wang J., Li J. F., Li Y., Yang H., Yu H. Y., Ma X. B., Yaer X. B., Liu L., Miao L. (2018). J. Mater. Chem. C.

[cit14] Lu Z. L., Zhang H. R., Lei W., Sinclair D. C., Reaney I. M. (2016). Chem. Mater..

[cit15] Ito M., Nagira T., Furumoto D., Katsuyama S., Nagai H. (2003). Scr. Mater..

[cit16] Nong N. V., Pryds N., Linderoth S., Ohtaki M. (2011). Adv. Mater..

[cit17] Romo-De-La-Cruz C. O., Chen Y., Liang L., Williams M., Song X. Y. (2020). Chem. Mater..

[cit18] Song X. Y., Navia S. A. P., Liang L., Boyle C., Romo-De-La-Cruz C. O., Jackson B., Hinerman A., Wilt M., Prucz J., Chen Y. (2018). ACS Appl. Mater. Interfaces.

[cit19] Zhou C. J., Lee Y. K., Yu Y., Byun S., Luo Z. Z., Lee H., Ge B. Z., Lee Y. L., Chen X. Q., Lee J. Y., Cojocaru-Mirédin O., Chang H., Im J., Cho S. P., Wuttig M., Dravid V. P., Kanatzidis M. G., Chung I. (2021). Nat. Mater..

[cit20] Wu H. J., Zhao L. D., Zheng F. S., Wu D., Pei Y. L., Tong X., Kanatzidis M. G., He J. Q. (2014). Nat. Commun..

[cit21] Luan Y. E., Li Y. M., Li Z., Zhang B. Y., Ou J. Z. (2025). Adv. Sci..

[cit22] Wan D., Bai S. L., Li X. D., Ai P., Guo W. R., Zhang J. Y., Tang S. W. (2024). Ceram. Int..

[cit23] Yu L. F., Chen A. L., Wang X. X., Wang H. M., Qin Z. Z., Qin G. Z. (2022). Phys. Rev. B.

[cit24] Sato N., Kuroda N., Nakamura S., Katsura Y., Kanazawa I., Kimura K., Mori T. (2021). J. Mater. Chem. A.

[cit25] Zhao L. D., Berardan D., Pei Y. L., Byl C., Pinsard-Gaudart L., Dragoe N. (2010). Appl. Phys. Lett..

[cit26] Pei Y. L., He J. Q., Li J. F., Li F., Liu Q. J., Pan W., Barreteau C., Berardan D., Dragoe N., Zhao L. D. (2013). NPG Asia Mater..

[cit27] Fan D. D., Liu H. J., Cheng L., Zhang J., Jiang P. H., Wei J., Liang J. H., Shi J. (2017). Phys. Chem. Chem. Phys..

[cit28] Maeda K., Takeiri F., Kobayashi G., Matsuishi S., Ogino H., Ida S., Mori T., Uchimoto Y., Tanabe S., Hasegawa T., Imanaka N., Kageyama H. (2022). Bull. Chem. Soc. Jpn..

[cit29] Liu Y., Zhao L. D., Zhu Y., Liu Y., Li F., Yu M., Liu D. B., Xu W., Lin Y. H., Nan C. W. (2016). Adv. Energy Mater..

[cit30] Einhorn M., Williamson B. A. D., Scanlon D. O. (2020). J. Mater. Chem. A.

[cit31] Rahim W., Skelton J. M., Scanlon D. O. (2021). J. Mater. Chem. A.

[cit32] Wang N., Li M. L., Xiao H. Y., Gao Z. B., Liu Z. J., Zu X. T., Li S. A., Qiao L. (2021). npj Comput. Mater..

[cit33] Flitcroft J. M., Althubiani A., Skelton J. M. (2024). JPhys Energy.

[cit34] Ai P., Wang H., Tang S. W., Yan T. Y., Bai S. L., Wan D., Guo W. R., Zhang P. F., Zheng T. (2025). J. Energy Chem..

[cit35] Nakada A., Kato D., Nelson R., Takahira H., Yabuuchi M., Higashi M., Suzuki H., Kirsanova M., Kakudou N., Tassel C., Yamamoto T., Brown C. M., Dronskowski R., Saeki A., Abakumov A., Kageyama H., Abe R. (2021). J. Am. Chem. Soc..

[cit36] Kresse G., Furthmuller J. (1996). Comput. Mater. Sci..

[cit37] Blochl P. E. (1994). Phys. Rev. B:Condens. Matter Mater. Phys..

[cit38] Kresse G., Joubert D. (1999). Phys. Rev. B:Condens. Matter Mater. Phys..

[cit39] Jaramillo J., Scuseria G. E., Ernzerhof M. (2003). J. Chem. Phys..

[cit40] Krukau A. V., Vydrov O. A., Izmaylov A. F., Scuseria G. E. (2006). J. Chem. Phys..

[cit41] Perdew J. P., Ruzsinszky A., Csonka G. I., Vydrov O. A., Scuseria G. E., Constantin L. A., Zhou X., Burke K. (2008). Phys. Rev. Lett..

[cit42] Pulay P. (1969). Mol. Phys..

[cit43] Perdew J. P., Burke K., Ernzerhof M. (1996). Phys. Rev. Lett..

[cit44] Furness J. W., Kaplan A. D., Ning J. L., Perdew J. P., Sun J. W. (2020). J. Phys. Chem. Lett..

[cit45] Ganose A., Jackson A., Scanlon D. (2018). J. Open Source Softw..

[cit46] Bradley C. J. and Cracknell A. P., The Mathematical Theory of Symmetry in Solids: Representation Theory for Point Groups and Space Groups, Clarendon Press, Oxford, 1972

[cit47] Ganose A. M., Park J., Faghaninia A., Woods-Robinson R., Persson K. A., Jain A. (2021). Nat. Commun..

[cit48] Spooner K. B., Ganose A. M., Leung W. W. W., Buckeridge J., Williamson B. A. D., Palgrave R. G., Scanlon D. O. (2021). Chem. Mater..

[cit49] Togo A., Tanaka I. (2015). Scr. Mater..

[cit50] Togo A., Chaput L., Tanaka I. (2015). Phys. Rev. B:Condens. Matter Mater. Phys..

[cit51] Milne C. J., Lightfoot P., Jorgensen J. D., Short S. (1995). J. Mater. Chem..

[cit52] Momma K., Izumi F. (2011). J. Appl. Crystallogr..

[cit53] Pallikara I., Flitcroft J. M., Skelton J. M. (2022). Solids.

[cit54] Spooner K. B., Einhorn M., Davies D. W., Scanlon D. O. (2024). J. Open Source Softw..

[cit55] Zhang X. X., Chang C., Zhou Y. M., Zhao L. D. (2017). Materials.

[cit56] Dou W. Z., Spooner K. B., Kavanagh S. R., Zhou M., Scanlon D. O. (2024). J. Am. Chem. Soc..

[cit57] Han D., Zhu B. N., Cai Z. H., Spooner K. B., Rudel S. S., Schnick W., Bein T., Scanlon D. O., Ebert H. (2024). Matter.

[cit58] Lindsay L., Broido D. A. (2011). Phys. Rev. B:Condens. Matter Mater. Phys..

[cit59] Lindsay L., Broido D. A., Mingo N. (2010). Phys. Rev. B:Condens. Matter Mater. Phys..

[cit60] Ren G. K., Wang S. Y., Zhou Z. F., Li X., Yang J., Zhang W. Q., Lin Y. H., Yang J. H., Nan C. W. (2019). Nat. Commun..

[cit61] Mehta R. J., Zhang Y. L., Karthik C., Singh B., Siegel R. W., Borca-Tasciuc T., Ramanath G. (2012). Nat. Mater..

[cit62] Zhu T. J., Xu Z. J., He J., Shen J. J., Zhu S., Hu L. P., Tritt T. M., Zhao X. B. (2013). J. Mater. Chem. A.

[cit63] Hu L. P., Zhu T. J., Liu X. H., Zhao X. B. (2014). Adv. Funct. Mater..

[cit64] Kim S. I., Lee K. H., Mun H. A., Kim H. S., Hwang S. W., Roh J. W., Yang D. J., Shin W. H., Li X. S., Lee Y. H., Snyder G. J., Kim S. W. (2015). Science.

[cit65] Biswas K., He J. Q., Blum I. D., Wu C. I., Hogan T. P., Seidman D. N., Dravid V. P., Kanatzidis M. G. (2012). Nature.

[cit66] Zhao L. D., He J. Q., Wu C. I., Hogan T. P., Zhou X. Y., Uher C., Dravid V. P., Kanatzidis M. G. (2012). J. Am. Chem. Soc..

[cit67] Zhao L. D., He J. Q., Hao S. Q., Wu C. I., Hogan T. P., Wolverton C., Dravid V. P., Kanatzidis M. G. (2012). J. Am. Chem. Soc..

[cit68] Zhang Q., Cao F., Liu W. S., Lukas K., Yu B., Chen S., Opeil C., Broido D., Chen G., Ren Z. F. (2012). J. Am. Chem. Soc..

[cit69] Zhao L. D., Hao S. Q., Lo S. H., Wu C. I., Zhou X. Y., Lee Y., Li H., Biswas K., Hogan T. P., Uher C., Wolverton C., Dravid V. P., Kanatzidis M. G. (2013). J. Am. Chem. Soc..

[cit70] Zhao L. D., Wu H. J., Hao S. Q., Wu C. I., Zhou X. Y., Biswas K., He J. Q., Hogan T. P., Uher C., Wolverton C., Dravid V. P., Kanatzidis M. G. (2013). Energy Environ. Sci..

[cit71] Korkosz R. J., Chasapis T. C., Lo S. H., Doak J. W., Kim Y. J., Wu C. I., Hatzikraniotis E., Hogan T. P., Seidman D. N., Wolverton C., Dravid V. P., Kanatzidis M. G. (2014). J. Am. Chem. Soc..

[cit72] Tan G. J., Shi F. Y., Hao S. Q., Zhao L. D., Chi H., Zhang X. M., Uher C., Wolverton C., Dravid V. P., Kanatzidis M. G. (2016). Nat. Commun..

[cit73] Hodges J. M., Hao S. Q., Grovogui J. A., Zhang X. M., Bailey T. P., Li X., Gan Z. H., Hu Y. Y., Uher C., Dravid V. P., Wolverton C., Kanatzidis M. G. (2018). J. Am. Chem. Soc..

[cit74] Olvera A. A., Moroz N. A., Sahoo P., Ren P., Bailey T. P., Page A. A., Uher C., Poudeu P. F. P. (2017). Energy Environ. Sci..

[cit75] Liu H. L., Shi X., Xu F. F., Zhang L. L., Zhang W. Q., Chen L. D., Li Q., Uher C., Day T., Snyder G. J. (2012). Nat. Mater..

[cit76] Yang L., Chen Z. G., Han G., Hong M., Zou Y. C., Zou J. (2015). Nano Energy.

[cit77] He Y., Lu P., Shi X., Xu F. F., Zhang T. S., Snyder G. J., Uher C., Chen L. D. (2015). Adv. Mater..

[cit78] Zhao K. P., Duan H. Z., Raghavendra N., Qiu P. F., Zeng Y., Zhang W. Q., Yang J. H., Shi X., Chen L. D. (2017). Adv. Mater..

[cit79] Nunna R., Qiu P. F., Yin M. J., Chen H. Y., Hanus R., Song Q. F., Zhang T. S., Chou M. Y., Agne M. T., He J. Q., Snyder G. J., Shi X., Chen L. D. (2017). Energy Environ. Sci..

[cit80] Zhao K. P., Zhu C. X., Qiu P. F., Blichfeld A. B., Eikeland E., Ren D. D., Iversen B. B., Xu F. F., Shi X., Chen L. D. (2017). Nano Energy.

[cit81] Zhao L. L., Islam S., Wang J., Cortie D. L., Wang X. G., Cheng Z. X., Wang J. Y., Ye N., Dou S. X., Shi X., Chen L. D., Snyder G. J., Wang X. L. (2017). Nano Energy.

[cit82] Li M., Cortie D. L., Liu J. X., Yu D. H., Islam S., Zhao L. L., Mitchell D. R. G., Mole R. A., Cortie M. B., Dou S. X., Wang X. L. (2018). Nano Energy.

[cit83] Zhang Q., Liao B. L., Lan Y. C., Lukas K., Liu W. S., Esfarjani K., Opeil C., Broido D., Chen G., Ren Z. F. (2013). Proc. Natl. Acad. Sci. U. S. A..

[cit84] Tan G. J., Shi F. Y., Hao S. Q., Chi H., Bailey T. P., Zhao L. D., Uher C., Wolverton C., Dravid V. P., Kanatzidis M. G. (2015). J. Am. Chem. Soc..

[cit85] Zhao L. D., Zhang X., Wu H. J., Tan G. J., Pei Y. L., Xiao Y., Chang C., Wu D., Chi H., Zheng L., Gong S. K., Uher C., He J. Q., Kanatzidis M. G. (2016). J. Am. Chem. Soc..

[cit86] Al Orabi R. A., Mechosky N. A., Hwang J., Kim W., Rhyee J. S., Wee D., Fornari M. (2016). Chem. Mater..

[cit87] Li W., Zheng L. L., Ge B. H., Lin S. Q., Zhang X. Y., Chen Z. W., Chang Y. J., Pei Y. Z. (2017). Adv. Mater..

[cit88] Hu L. P., Zhang Y., Wu H. J., Li J. Q., Li Y., McKenna M., He J., Liu F. S., Pennycook S. J., Zeng X. R. (2018). Adv. Energy Mater..

[cit89] Zhou Z. W., Yang J. Y., Jiang Q. H., Lin X. S., Xin J. W., Basit A., Hou J. D., Sun B. Y. (2018). Nano Energy.

[cit90] Banik A., Ghosh T., Arora R., Dutta M., Pandey J., Acharya S., Soni A., Waghmare U. V., Biswas K. (2019). Energy Environ. Sci..

[cit91] Perumal S., Roychowdhury S., Biswas K. (2016). Inorg. Chem. Front..

[cit92] Hong M., Chen Z. G., Yang L., Zou Y. C., Dargusch M. S., Wang H., Zou J. (2018). Adv. Mater..

[cit93] Li J., Zhang X. Y., Wang X., Bu Z. L., Zheng L. T., Zhou B. Q., Long F., Chen Y., Pei Y. Z. (2018). J. Am. Chem. Soc..

[cit94] Liu Z. H., Sun J. F., Mao J., Zhu H. T., Ren W. Y., Zhou J. C., Wang Z. M., Singh D. J., Sui J. H., Chu C. W., Ren Z. F. (2018). Proc. Natl. Acad. Sci. U. S. A..

[cit95] Zhang X. Y., Li J., Wang X., Chen Z. W., Mao J. J., Chen Y., Pei Y. Z. (2018). J. Am. Chem. Soc..

[cit96] Zheng Z., Su X. L., Deng R. G., Stoumpos C., Xie H. Y., Liu W., Yan Y. G., Hao S. Q., Uher C., Wolverton C., Kanatzidis M. G., Tang X. F. (2018). J. Am. Chem. Soc..

[cit97] Srinivasan B., Gellé A., Gucci F., Boussard-Pledel C., Fontaine B., Gautier R., Halet J. F., Reece M. J., Bureau B. (2019). Inorg. Chem. Front..

[cit98] Hong M., Wang Y., Feng T. L., Sun Q., Xu S. D., Matsumura S., Pantelides S. T., Zou J., Chen Z. G. (2019). J. Am. Chem. Soc..

[cit99] Du B. L., Li H., Xu J. J., Tang X. F., Uher C. (2010). Chem. Mater..

[cit100] Hong M., Chen Z. G., Yang L., Liao Z. M., Zou Y. C., Chen Y. H., Matsumura S., Zou J. (2018). Adv. Energy Mater..

[cit101] Chen X., Zhou J. S., Goodenough J. B., Shi L. (2015). J. Mater. Chem. C.

[cit102] Muthiah S., Singh R. C., Pathak B. D., Avasthi P. K., Kumar R., Kumar A., Srivastava A. K., Dhar A. (2018). Nanoscale.

[cit103] Gao Z. P., Xiong Z. W., Li J., Lu C. J., Zhang G. H., Zeng T., Ma Y. J., Ma G. H., Zhang R. Z., Chen K., Zhang T., Liu Y., Yang J., Cao L. H., Jin K. (2019). J. Mater. Chem. A.

[cit104] Kuo Y. K., Ramachandran B., Lue C. S. (2014). Front. Chem..

[cit105] He Y., Day T., Zhang T. S., Liu H. L., Shi X., Chen L. D., Snyder G. J. (2014). Adv. Mater..

[cit106] Meng Q. L., Kong S., Huang Z. W., Zhu Y. H., Liu H. C., Lu X. W., Jiang P., Bao X. H. (2016). J. Mater. Chem. A.

[cit107] Tang H. C., Sun F. H., Dong J. F., Asfandiyar, Zhuang H. L., Pan Y., Li J. F. (2018). Nano Energy.

[cit108] Ge Z. H., Zhang Y. X., Song D. S., Chong X. Y., Qin P., Zheng F. S., Feng J., Zhao L. D. (2018). J. Mater. Chem. A.

[cit109] Yang H. Q., Wang X. Y., Wu H., Zhang B., Xie D. D., Chen Y. J., Lu X., Han X. D., Miao L., Zhou X. Y. (2019). J. Mater. Chem. C.

[cit110] Deng T. T., Qiu P. F., Xing T., Zhou Z. Y., Wei T. R., Ren D. D., Xiao J., Shi X., Chen L. D. (2021). J. Mater. Chem. A.

[cit111] Guélou G., Powell A. V., Vaqueiro P. (2015). J. Mater. Chem. C.

[cit112] Kikuchi Y., Bouyrie Y., Ohta M., Suekuni K., Aihara M., Takabatake T. (2016). J. Mater. Chem. A.

[cit113] Zheng Y., Slade T. J., Hu L., Tan X. Y., Luo Y. B., Luo Z. Z., Xu J. W., Yan Q. Y., Kanatzidis M. G. (2021). Chem. Soc. Rev..

[cit114] Li J., Sui J. H., Pei Y. L., Barreteau C., Berardan D., Dragoe N., Cai W., He J. Q., Zhao L. D. (2012). Energy Environ. Sci..

[cit115] Liang S. J., Xu J. T., Noudem J. G., Wang H. X., Tan X. J., Liu G. Q., Shao H. Z., Yu B., Yue S., Jiang J. (2018). J. Mater. Chem. A.

[cit116] Liu J., Wang P., Wang M. Y., Xu R., Zhang J., Liu J. Z., Li D., Liang N. N., Du Y. W., Chen G., Tang G. D. (2018). Nano Energy.

[cit117] Shi X. L., Zheng K., Hong M., Liu W. D., Moshwan R., Wang Y., Qu X. L., Chen Z. G., Zou J. (2018). Chem. Sci..

[cit118] Shi X. L., Wu A., Feng T. L., Zheng K., Liu W. D., Sun Q., Hong M., Pantelides S. T., Chen Z. G., Zou J. (2019). Adv. Energy Mater..

[cit119] Luo Y. B., Cai S. T., Hua X., Chen H. J., Liang Q. H., Du C. F., Zheng Y., Shen J. H., Xu J. W., Wolverton C., Dravid V. P., Yan Q. Y., Kanatzidis M. G. (2019). Adv. Energy Mater..

[cit120] Maintz S., Deringer V. L., Tchougréeff A. L., Dronskowski R. (2016). J. Comput. Chem..

[cit121] Fan H., Wu H., Lindsay L., Hu Y. J. (2019). Phys. Rev. B.

