# Supplementary material for: Excellent thermoelectric performance of Bi2MO4Cl (M = Y, La, and Bi) derived from ultra-low lattice thermal conductivity
Source: J Mater Chem A Mater. 2025 Sep 22;13(41):35507–20. doi: 10.1039/d5ta05523g (PMC12451671; doi:10.1039/d5ta05523g)
Supplement: TA-013-D5TA05523G-s001 [file TA-013-D5TA05523G-s001.pdf]

Supplementary Information (SI) for Journal of Materials Chemistry A.
This journal is © The Royal Society of Chemistry 2025

Supplementary Information

Excellent Thermoelectric Performance of Bi_2MO_4Cl (M = Y, La, and Bi) Derived from Ultra-Low Lattice Thermal Conductivity

Shipeng Bi[a], Christopher N. Savory[b], Alexander G. Squires[c], Dan Han[d], Kieran B. Spooner[d] and David O. Scanlon[c]

[a] *Department of Chemistry, University College London, 20 Gordon Street, London WC1H 0AJ, United Kingdom*

[b] *Department of Chemistry, Swansea University, Singleton Park, Swansea SA2 8PP, United Kingdom*

[c] *School of Chemistry, University of Birmingham, Edgbaston, Birmingham B15 2TT, United Kingdom*

[d] *School of Materials Science and Engineering, Jilin University, Changchun 130012, China*

1 Energy cutoff and *k*-point mesh convergence tests

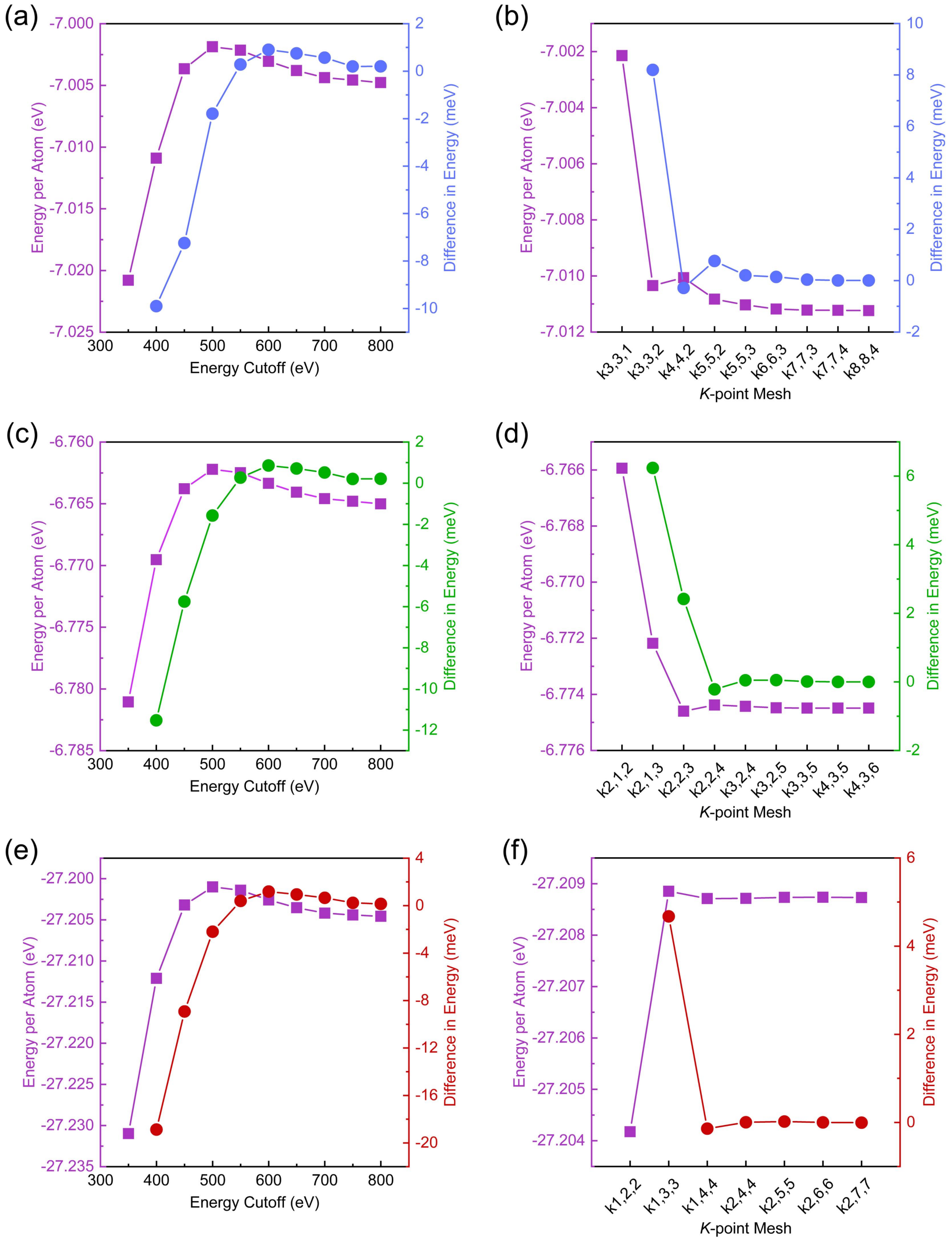

Figure S1 Convergence tests of the energy cutoffs and the *k*-point meshes for Bi_2YO_4Cl ((a) and (b)), Bi_2LaO_4Cl ((c) and (d)), and Bi_3O_4Cl ((e) and (f)) applied to the conventional unit cells. The square symbols represent the average energy per atom of the crystals, while the circular symbols represent the energy difference between consecutive tests. The energy cutoffs of 550 eV for Bi_2YO_4Cl and Bi_2LaO_4Cl, and 700 eV for Bi_3O_4Cl were chosen to be applied to the calculations. The *k*-point meshes of 6 × 6 × 3, 2 × 2 × 4, 1 × 4 × 4 were found to converge for Bi_2YO_4Cl, Bi_2LaO_4Cl, and Bi_3O_4Cl, respectively.

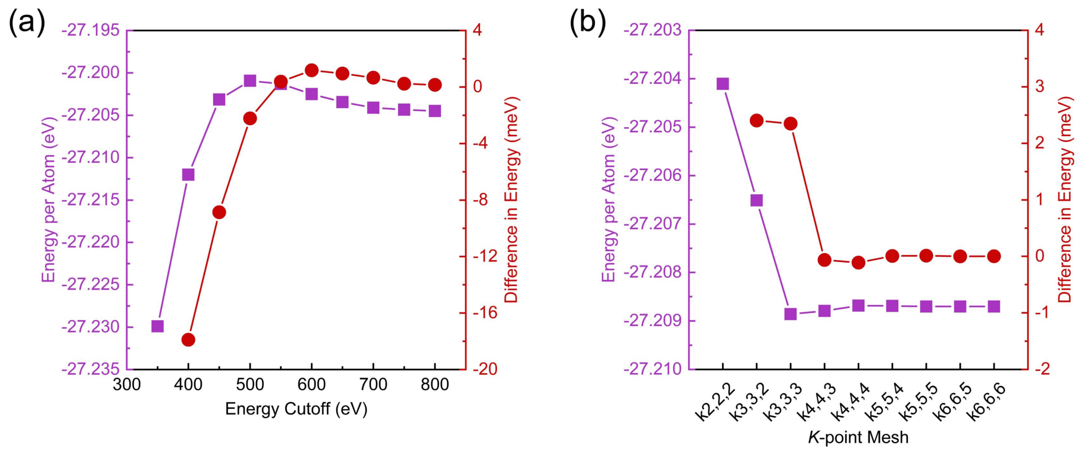

Figure S2 Convergence tests of (a) the energy cutoff and (b) the *k*-point mesh for Bi_3O_4Cl using the primitive unit cell. The square symbols represent the average energy per atom of the crystal, while the circular symbols represent the energy difference between consecutive tests. An energy cutoff of 700 eV and a *k*-point mesh at 4×4×3 were selected for the calculations.

2 Phonon dispersions of Bi_3O_4Cl

We optimised the structure of Bi_3O_4Cl using four different functionals and used the optimised structures to construct supercells with an expanded mesh of 1 × 2 × 2 to obtain the phonon dispersions (Figure S3). By comparing the optimised lattice parameters with the experimental data (Table S1), we found that the PBE functional significantly overestimates the lattice parameters, especially in the b and c directions. Additionally, the PBEsol functional could not be used for subsequent third-order force constants (FCs) calculations because the structure it optimised is not dynamically stable. Due to the enormous computational expense, using the HSE06 functional to perform thousands of self-consistent calculations is impractical. The lattice parameters optimised with the r^2SCAN functional matched the experimental values well, and therefore this functional was chosen to evaluate the third-order FCs of Bi_3O_4Cl.

Table S1 Lattice parameters of Bi_3O_4Cl optimised using four different functionals. The percentage differences from the experimental parameters[1] are given in parentheses. The experimental lattice parameters for the *C2/c* structure of Bi_3O_4Cl were converted from those of the *I2/a* structure.

	a (Å)	*b* (Å)	*c* (Å)
Nakada et al. (exp.[1])	19.28	5.65	5.69
PBE	19.75 (2.44%)	5.80 (2.65%)	5.82 (2.28%)
PBEsol	19.14 (-0.73%)	5.66 (0.18%)	5.67 (-0.35%)
r^2SCAN	19.45 (0.88%)	5.70 (0.88%)	5.73 (0.70%)
HSE06	19.54 (1.35%)	5.72 (1.24%)	5.76 (1.23%)

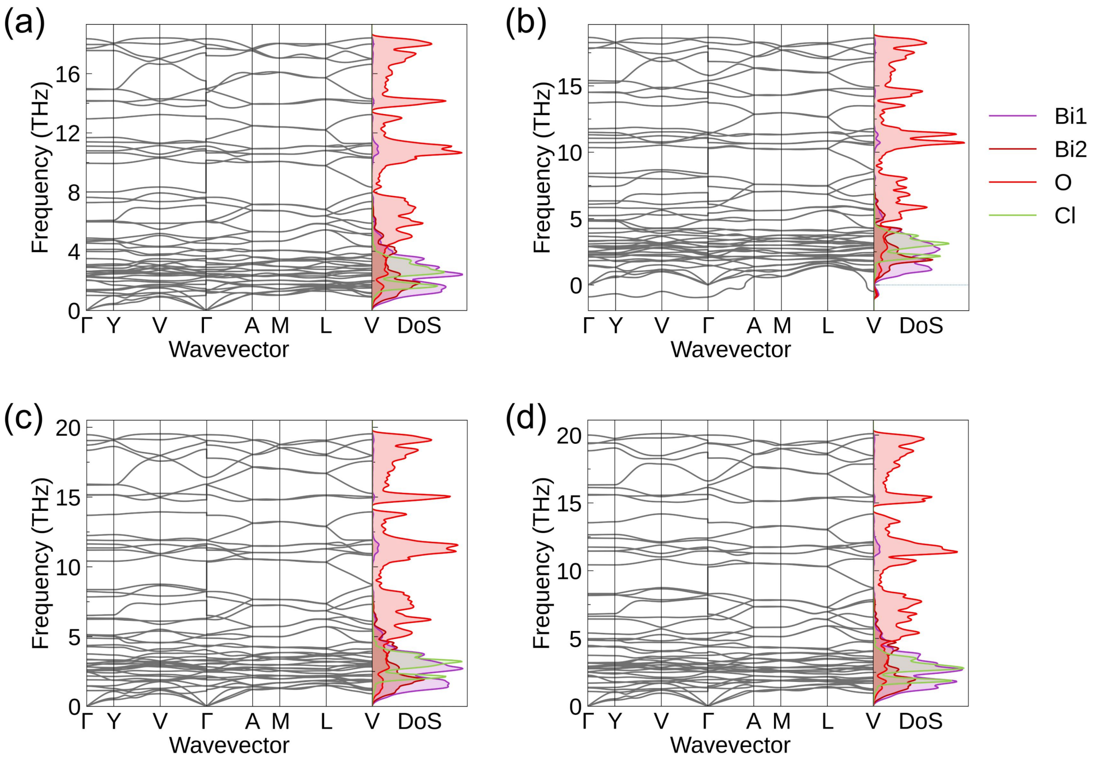

Figure S3 Phonon dispersions and atom-projected phonon densities of states (DoS) of Bi_3O_4Cl calculated using the (a) PBE, (b) PBEsol, (c) r^{2}SCAN, and (d) HSE06 functionals.

3 AMSET setting

Bi_2YO_4Cl

$$\text{Deformation potential tensor (CBM) (eV)} = \begin{bmatrix} 4.15 & 0.18 & 0.07 \\ 0.18 & 4.15 & 0.07 \\ 0.07 & 0.07 & 4.88 \end{bmatrix}$$

$$\text{Deformation potential tensor (VBM) (eV)} = \begin{bmatrix} 2.50 & 0.12 & 0.01 \\ 0.12 & 1.64 & 0.01 \\ 0.01 & 0.01 & 1.18 \end{bmatrix}$$

$$\text{High-frequency dielectric constant } (\epsilon_0) = \begin{bmatrix} 4.76 & 0 & 0 \\ 0 & 4.76 & 0 \\ 0 & 0 & 4.30 \end{bmatrix}$$

$$\text{Static dielectric constant } (\epsilon_0) = \begin{bmatrix} 27.03 & 0 & 0 \\ 0 & 27.03 & 0 \\ 0 & 0 & 16.04 \end{bmatrix}$$

$$\text{Elastic constant (GPa)} = \begin{bmatrix} 178.3 & 96.7 & 32.6 & 0 & 0 & 0 \\ 96.7 & 178.3 & 32.6 & 0 & 0 & 0 \\ 32.6 & 32.6 & 92.4 & 0 & 0 & 0 \\ 0 & 0 & 0 & 23.1 & 0 & 0 \\ 0 & 0 & 0 & 0 & 23.3 & 0 \\ 0 & 0 & 0 & 0 & 0 & 88.5 \end{bmatrix}$$

Polar optical phonon frequency (THz) = 7.67

Bi_2LaO_4Cl

$$\text{Deformation potential tensor (CBM) (eV)} = \begin{bmatrix} 2.42 & 0.13 & 0.10 \\ 0.13 & 4.04 & 0.01 \\ 0.10 & 0.01 & 4.95 \end{bmatrix}$$

$$\text{Deformation potential tensor (VBM) (eV)} = \begin{bmatrix} 0.16 & 0.09 & 0.03 \\ 0.09 & 0.33 & 4.03 \\ 0.03 & 4.03 & 0.47 \end{bmatrix}$$

$$\text{High-frequency dielectric constant } (\epsilon_0) = \begin{bmatrix} 3.98 & 0 & 0.01 \\ 0 & 4.29 & 0 \\ 0.01 & 0 & 4.26 \end{bmatrix}$$

$$\text{Static dielectric constant } (\epsilon_0) = \begin{bmatrix} 16.36 & 0 & -0.75 \\ 0 & 23.90 & 0 \\ -0.75 & 0 & 26.82 \end{bmatrix}$$

$$\text{Elastic constant (GPa)} = \begin{bmatrix} 79.4 & 37.6 & 30.2 & 0 & -0.8 & 0 \\ 37.6 & 101.3 & 49.1 & 0 & 0.9 & 0 \\ 30.2 & 49.1 & 152.8 & 0 & -4.5 & 0 \\ 0 & 0 & 0 & 36.2 & 0 & -0.2 \\ -0.8 & 0.9 & -4.5 & 0 & 15.1 & 0 \\ 0 & 0 & 0 & -0.2 & 0 & 11.0 \end{bmatrix}$$

Polar optical phonon frequency (THz) = 7.92

Bi_3O_4Cl

Deformation potential tensor (CBM) (eV) = $\begin{bmatrix} 0.63 & 0.09 & 3.48 \\ 0.09 & 4.73 & 0.19 \\ 3.48 & 0.19 & 5.11 \end{bmatrix}$

Deformation potential tensor (VBM) (eV) = $\begin{bmatrix} 0.39 & 0.09 & 1.24 \\ 0.09 & 1.86 & 0.01 \\ 1.24 & 0.01 & 1.91 \end{bmatrix}$

High-frequency dielectric constant (ϵ_0) = $\begin{bmatrix} 4.33 & 0 & -0.04 \\ 0 & 4.40 & 0 \\ -0.04 & 0 & 4.50 \end{bmatrix}$

Static dielectric constant (ϵ_0) = $\begin{bmatrix} 24.29 & 0 & -2.53 \\ 0 & 19.49 & 0 \\ -2.53 & 0 & 28.05 \end{bmatrix}$

Elastic constant (GPa) = $\begin{bmatrix} 62.4 & 27.4 & 20.2 & 0 & 1.6 & 0 \\ 27.4 & 83.6 & 18.8 & 0 & 3.6 & 0 \\ 20.2 & 18.8 & 87.3 & 0 & -12.3 & 0 \\ 0 & 0 & 0 & 23.2 & 0 & -2.3 \\ 1.6 & 3.6 & -12.3 & 0 & 18.4 & 0 \\ 0 & 0 & 0 & -2.3 & 0 & 21.6 \end{bmatrix}$

Polar optical phonon frequency (THz) = 7.47

4 Convergence tests of interpolation mesh for electronic transport properties

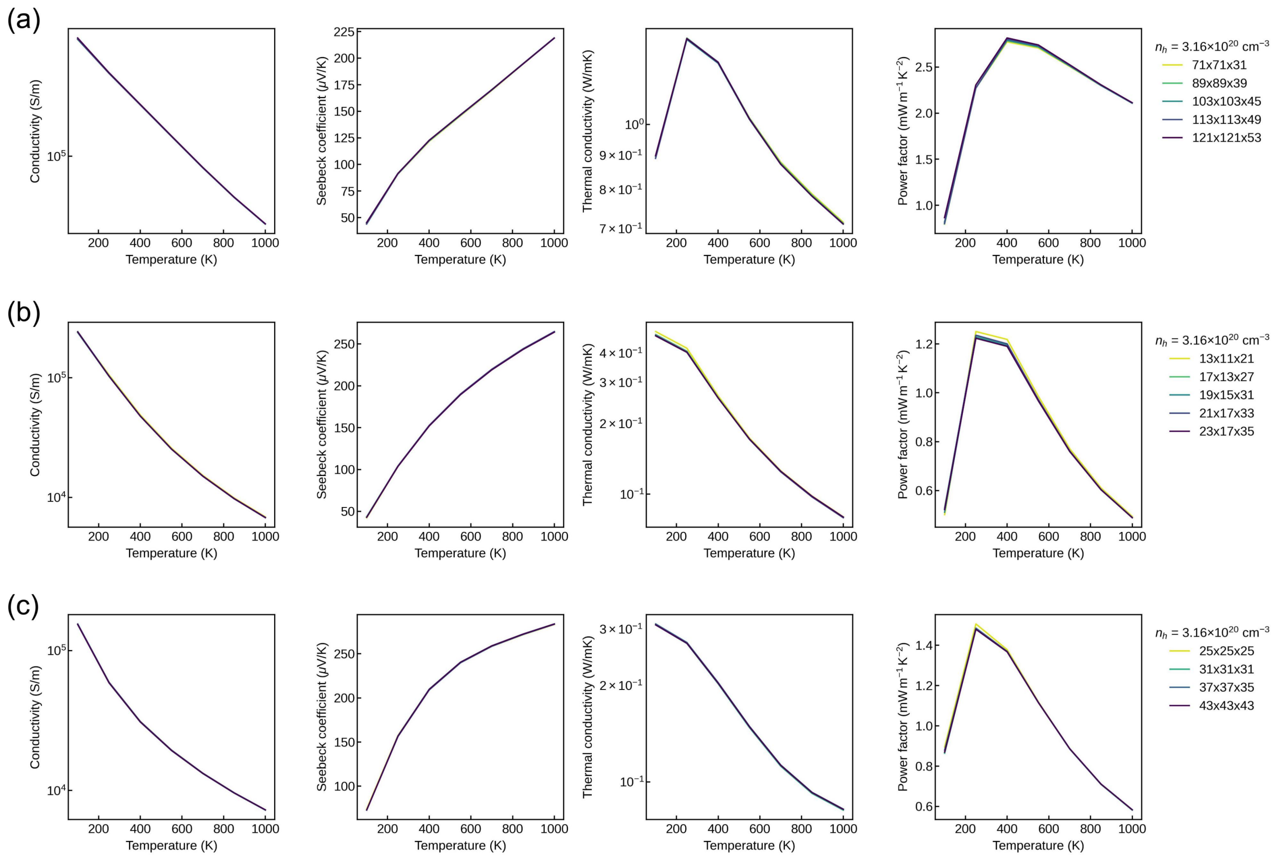

Figure S4 Electronic transport properties for (a) Bi_2YO_4Cl, (b) Bi_2LaO_4Cl and (c) Bi_3O_4Cl as a function of temperature, calculated using different interpolated meshes.

5 Phonon supercell mesh convergence tests

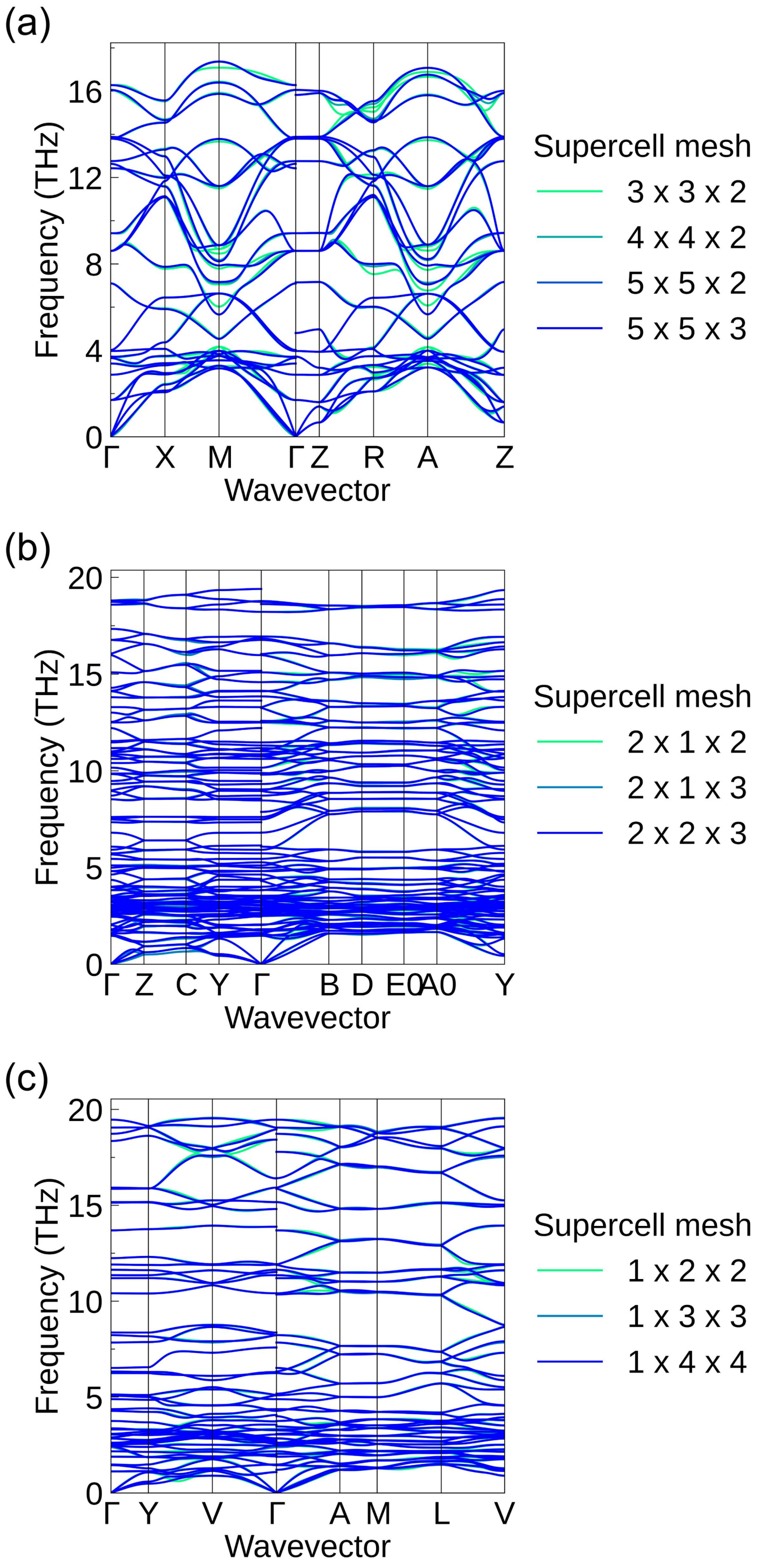

Figure S5 Phonon dispersions corresponding to different supercell meshes for (a) Bi_2YO_4Cl, (b) Bi_2LaO_4Cl, and (c) Bi_3O_4Cl, plotted using ThermoParser.[2] The high-symmetry path is based on the Bradley-Cracknell formalism.[3]

6 Convergence tests of lattice thermal conductivity with respect to *q*-point sampling mesh

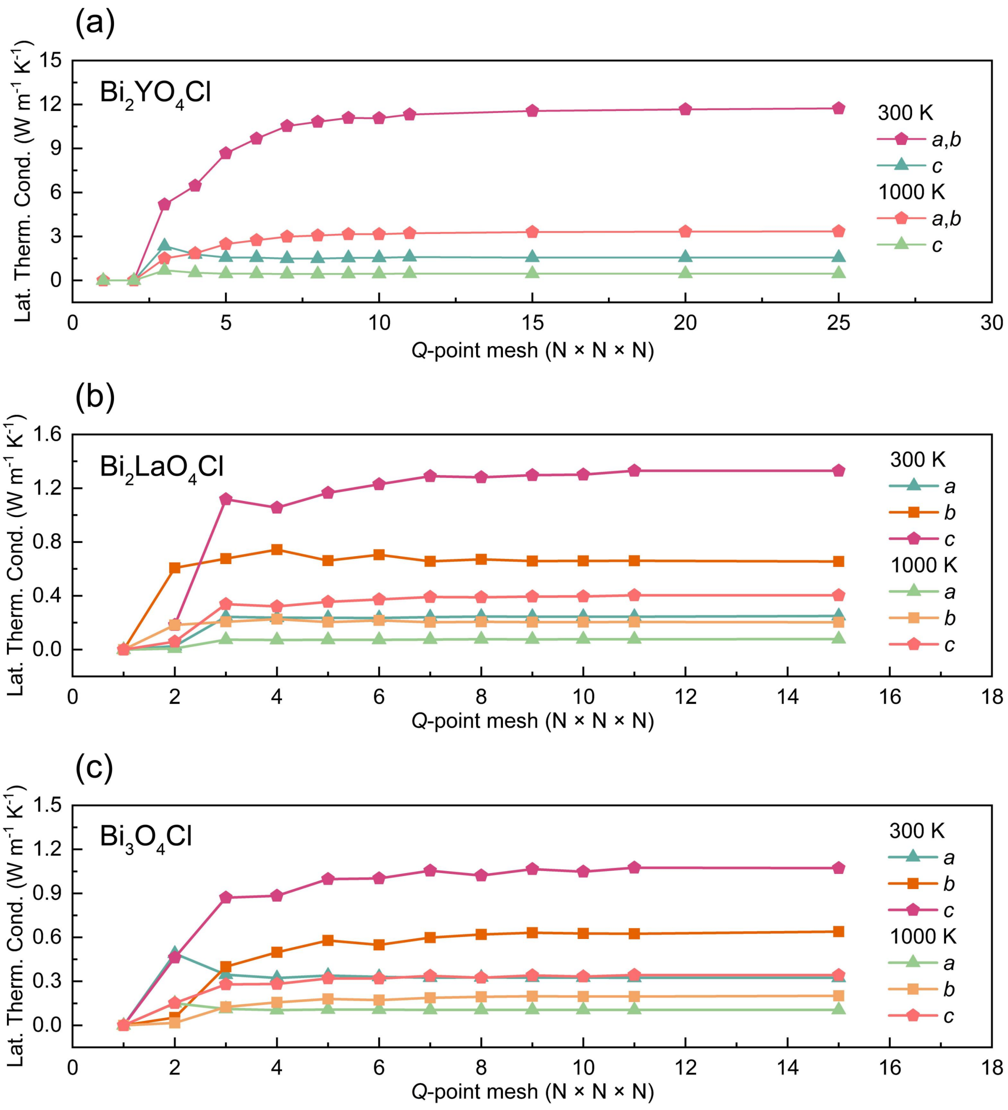

Figure S6 *Q*-point sampling mesh tests of the lattice thermal conductivity for (a) Bi_2YO_4Cl, (b) Bi_2LaO_4Cl, and (c) Bi_3O_4Cl.

7 ***P4/mmm* and *P2₁/c* structures of Bi_2LaO_4Cl**

We calculated the energies of two different space group structures (*P4/mmm* structure and $P2_1/c$ structure) separately and found that the *P4/mmm* structure has an energy of -6.644 eV per atom, while the $P2_1/c$ structure has an energy of -6.625 eV per atom. The lower energy indicates that the *P4/mmm* structure is the more stable. However, based on the free energy as a function of temperature (Figure S7), a crossover of the two free energy curves occurs at ~65 K, implying that a phase transition occurs near 65 K—i.e., the structure with the lower free energy is transformed from *P4/mmm* to $P2_1/c$. Given that our study focuses mainly on the *ZT* values in the range of 100-1000 K, the subsequent discussion will concentrate on the properties of the $P2_1/c$ structure.

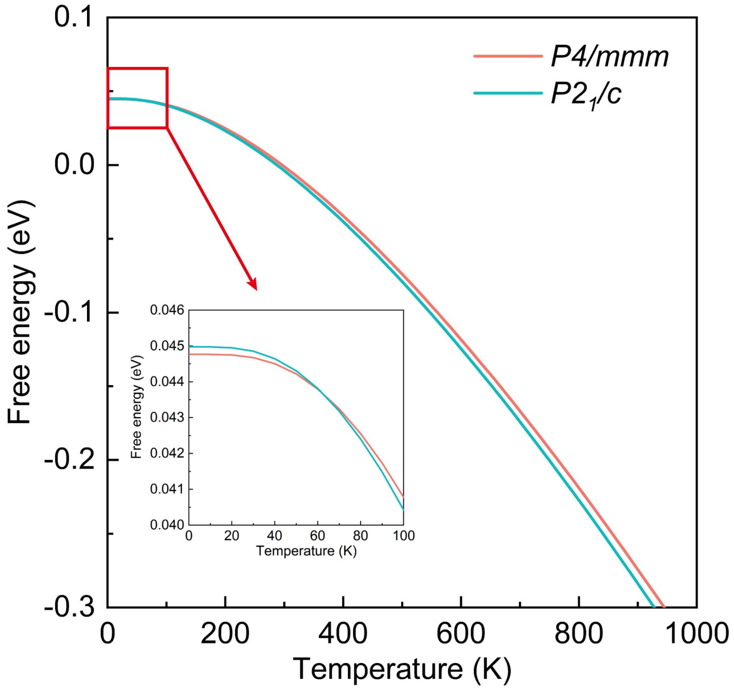

Figure S7 Temperature-dependent free energies of the *P4/mmm* and $P2_1/c$ structures of Bi_2LaO_4Cl.

8 N-type electronic transport properties and *ZT* values of Bi_2MO_4Cl (M = Y, La, and Bi)

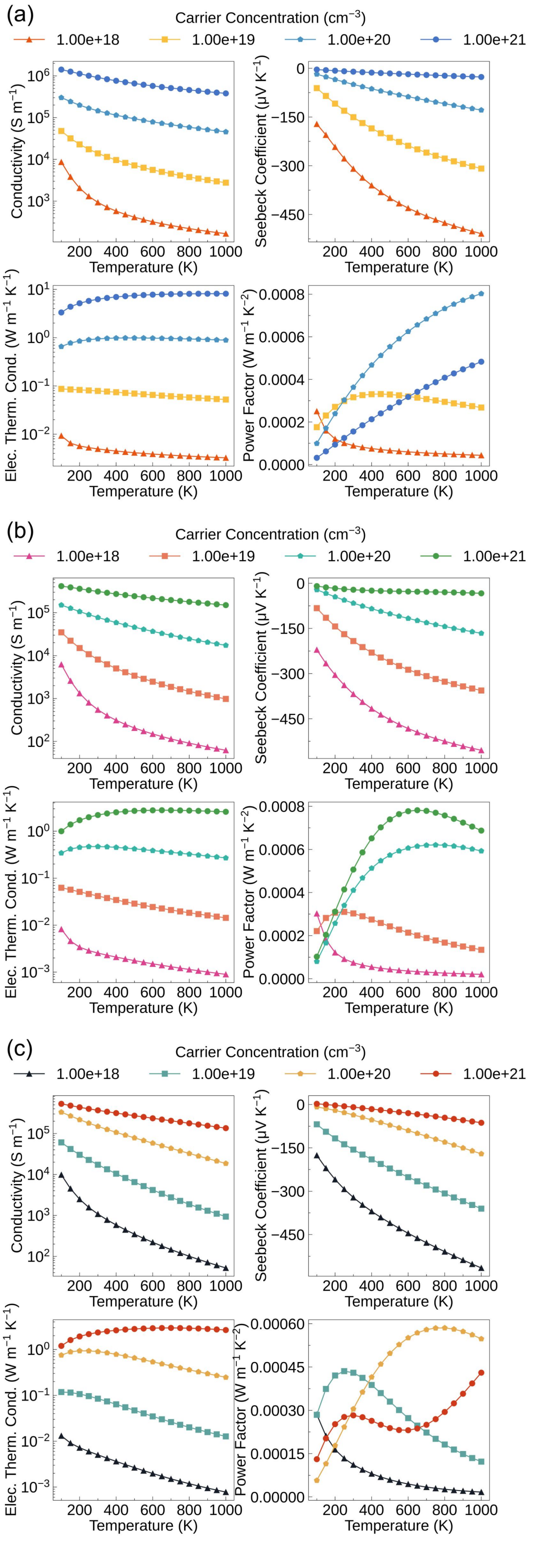

Figure S8 Calculated n-type electronic transport properties as a function of temperature for (a) Bi_2YO_4Cl, (b) Bi_2LaO_4Cl and (c) Bi_3O_4Cl with four different carrier concentrations.

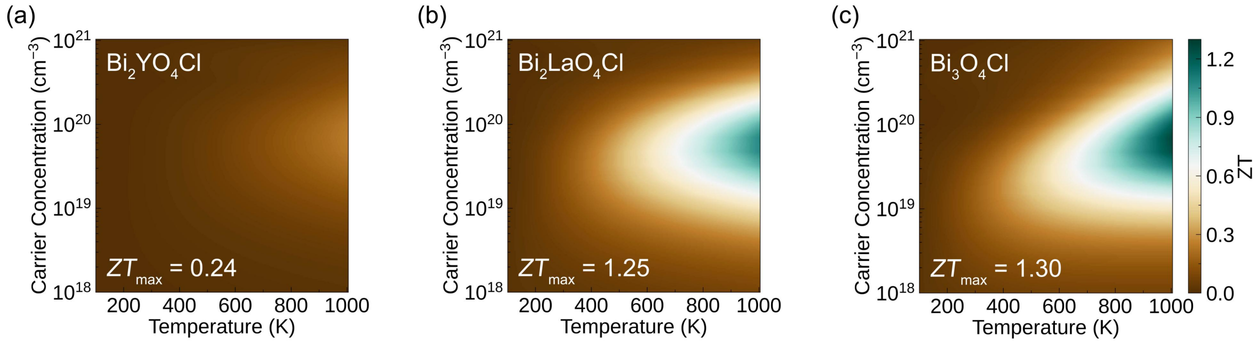

Figure S9 Predicted n-type ZT of (a) Bi_2YO_4Cl, (b) Bi_2LaO_4Cl and (c) Bi_3O_4Cl. This analysis was conducted using ThermoParser.[2]

Table S2 Predicted n-type maximum ZT values at 1000 K for Bi_2MO_4Cl (M = Y, La, and Bi) with the corresponding carrier concentration (n), lattice thermal conductivity (κ_l), power factor (PF) and electronic thermal conductivity (κ_e).

System	Direction	n (cm^{-3})	max ZT	κ_l (W m^{-1} K^{-1})	PF (μW m^{-1} K^{-2})	κ_e (W m^{-1} K^{-1})
Bi_2YO_4Cl	*a*, *b*	-1.00×10^{20}	0.23	3.30	1040	1.13
	c	-4.64×10^{19}	0.52	0.46	326	0.17
	Average	-4.64×10^{19}	0.24	2.35	651	0.36
Bi_2LaO_4Cl	*a*	-4.64×10^{19}	0.69	0.077	63.8	0.017
	b	-4.64×10^{19}	1.30	0.20	367	0.082
	c	-6.81×10^{19}	1.35	0.40	1000	0.34
	Average	-6.81×10^{19}	1.25	0.23	498	0.17
Bi_3O_4Cl	*a*	-4.64×10^{19}	0.98	0.11	166	0.059
	b	-6.81×10^{19}	1.48	0.20	548	0.17
	c	-6.81×10^{19}	1.27	0.33	659	0.19
	Average	-6.81×10^{19}	1.30	0.21	469	0.15

9 Scattering rates

Figure S10 shows the p-type average scattering rates for Bi_2MO_4Cl (M = Y, La, and Bi), and Figure S11 shows the n-type average scattering rates for these compounds. Here, we mainly consider acoustic deformation potential (ADP), ionic impurity (IMP), and polar optical phonon (POP) scattering. POP scattering refers to the process in which charge carriers interact with polar optical phonons. The relative vibration of atoms (which produces optical phonons) induces a change in the local electric field, which in turn affects the motion of carriers, altering their energy and momentum, which results in the carriers being scattered. ADP scattering is a scattering mechanism in which charge carriers interact with acoustic phonons. The collective vibration of atoms (which produces acoustic phonons) is accompanied by small deformations, leading to potential fluctuations. The carriers are subjected to these potential fluctuations, resulting in changes in their momentum and hence scattering. ADP scattering and POP scattering show a strong correlation with temperature because more phonons are excited as the temperature increases. IMP scattering is a scattering mechanism that occurs when charge carriers interact with impurity ions in a material. The charged impurity ions affect the motion of carriers through the Coulomb force, which deflects their trajectories and exhibits the scattering phenomenon. IMP scattering rates increase with carrier concentration but are weakly temperature dependent. At low temperatures, IMP scattering contributes most to the total scattering, and as the temperature increases, POP scattering gradually dominates (Figure S10(a), (d), (g) and Figure S11(a), (d), (g)). The trends of IMP and POP scattering are opposite when the carrier concentration is varied: at low carrier concentrations, the effect of POP scattering on the total scattering is more significant, while at high carrier concentration, both IMP and POP scattering play a non-negligible role in the total scattering (Figure S10(b), (e), (h) and Figure S11(b), (e), (h)). ADP scattering always contributes less to the total scattering. In the three compounds, Bi_2YO_4Cl has the lowest total scattering rates, which is mainly attributed to its larger high-frequency dielectric constant (due to the smaller bandgap), which helps to achieve higher conductivity. For all three compounds, IMP scattering exhibits the highest scattering rates near the edge of the energy band under maximised *ZT* values, while POP scattering rates are higher at higher energy states (Figure S10(c), (f), (i) and Figure S11(c), (f), (i)).

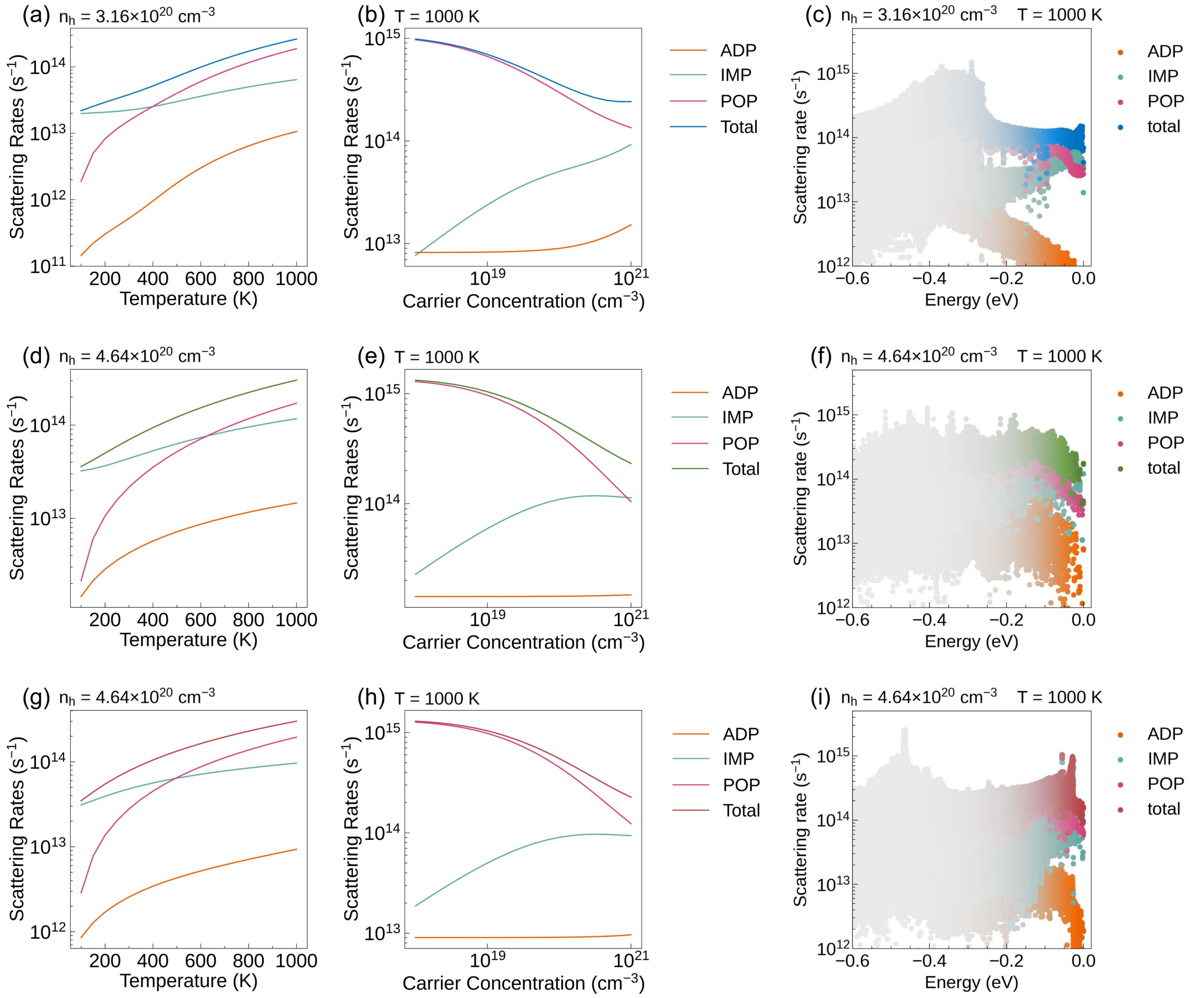

Figure S10 P-type average scattering rates of (a)-(c) Bi_2YO_4Cl, (d)-(e) Bi_2LaO_4Cl and (g)-(i) Bi_3O_4Cl with carrier concentrations and temperature fixed at the conditions corresponding to the predicted maximum *ZT* values. The average scattering rates are plotted as a function of (a), (d), and (g) temperature, (b), (e), and (h) carrier concentration and (c), (f), and (i) energy with respect to the VBM. In (c), (f) and (i), the colour intensity reflects the availability of carrier scattering channels via the band occupancies (and thus the weighted effect of the scattering rates on the overall mobility), given by the derivative of the Fermi-Dirac distribution function.

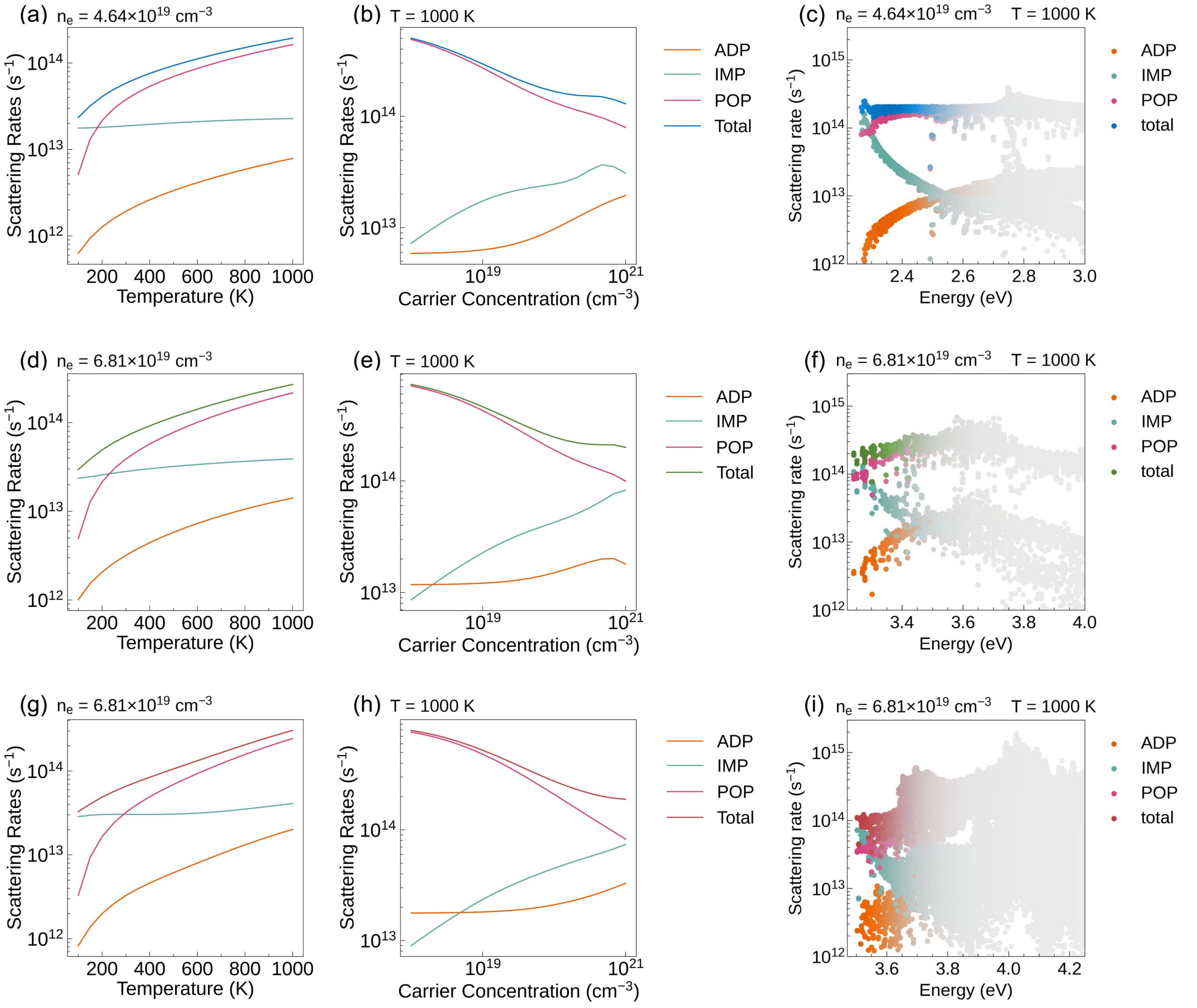

Figure S11 N-type average scattering rates of (a)-(c) Bi_2YO_4Cl, (d)-(e) Bi_2LaO_4Cl and (g)-(i) Bi_3O_4Cl with carrier concentrations and temperature fixed at the conditions corresponding to the predicted maximum *ZT* values. The average scattering rates are plotted as a function of (a), (d), and (g) temperature, (b), (e), and (h) carrier concentration and (c), (f), and (i) energy with respect to the CBM. In (c), (f) and (i), the colour intensity reflects the availability of carrier scattering channels via the band occupancies (and thus the weighted effect of the scattering rates on the overall mobility), given by the derivative of the Fermi-Dirac distribution function.

10 Heat-capacity-weighted root-mean-square (RMS) Grüneisen parameter

$$\gamma_{rms} = \sqrt{\frac{\sum_\lambda C_\lambda \gamma_\lambda^2}{\sum_\lambda C_\lambda}} \tag{S1}$$

where γ_{rms} is defined as the heat-capacity-weighted root-mean-square (RMS) Grüneisen parameter, C_λ is the heat capacity of the phonon mode λ, and γ_λ is the mode Grüneisen parameter. This equation reflects the overall effective anharmonicity, characterised by the heat-capacity-weighted RMS form.

In previous work, the average Grüneisen parameter could be obtained by taking the arithmetic mean of the Grüneisen parameters of the acoustic modes,[4] or by calculating the heat-capacity-weighted average of all phonon modes:[5]

$$\gamma = \frac{\sum_\lambda C_\lambda \gamma_\lambda}{\sum_\lambda C_\lambda} \tag{S2}$$

the latter is a more suitable approach for describing properties such as thermal expansion. However, for the system studied in this work, the former method is not applicable. This is because the acoustic and low-frequency optical branches are strongly mixed (Figure 3), making it difficult to clearly distinguish between them, whereas Phonopy assigns phonon branches solely based on frequency. If the position of the acoustic branch is determined solely based on frequency, this will inevitably lead to the incorrect identification of optical-mode Grüneisen parameters as acoustic-mode Grüneisen parameters, resulting in erroneous results.

For equation (S2), we replace γ with γ^2 and then take the square root on the right-hand side. The purpose of this modification is twofold: on the one hand, to eliminate the cancellation effect caused by the sign of γ, and on the other hand, to account for the relationship between the scattering rate and the Grüneisen parameter. In different phonon modes, the Grüneisen parameter γ_λ can take either positive or negative values. A positive γ_λ usually corresponds to modes that soften upon lattice expansion, while a negative γ_λ corresponds to modes that harden upon lattice expansion. If equation (S2) is directly adopted, the contributions from positive and negative γ_λ may cancel each other out, leading to an underestimation of the actual anharmonicity of the system. Such cancellation is reasonable in the description of thermal expansion, since it inherently relies on the combined contributions of positive and negative values; however, it is not suitable for explaining lattice thermal conductivity. At room temperature and above, lattice thermal conductivity is usually limited mainly by Umklapp scattering, in which the scattering rate is proportional to the square of the Grüneisen parameter ($\tau_U^{-1} \propto \gamma^2$).[4,6] Therefore, regardless of the sign of γ_λ, its contribution to scattering is always positive. If a linear weighted average were still used, the cancellation of

positive and negative values would underestimate the actual scattering intensity, leading to a deviation in the interpretation of lattice thermal conductivity.

In the calculation of the heat-capacity-weighted RMS Grüneisen parameter, the mode-dependent heat capacity C_λ at 300 K was employed. We restricted the phonon-mode range to those whose cumulative contribution to the lattice thermal conductivity accounts for 0-80% of the total, and calculated the heat-capacity-weighted RMS Grüneisen parameter within this range to ensure that the results reflect the anharmonicity of the phonons dominating heat transport.

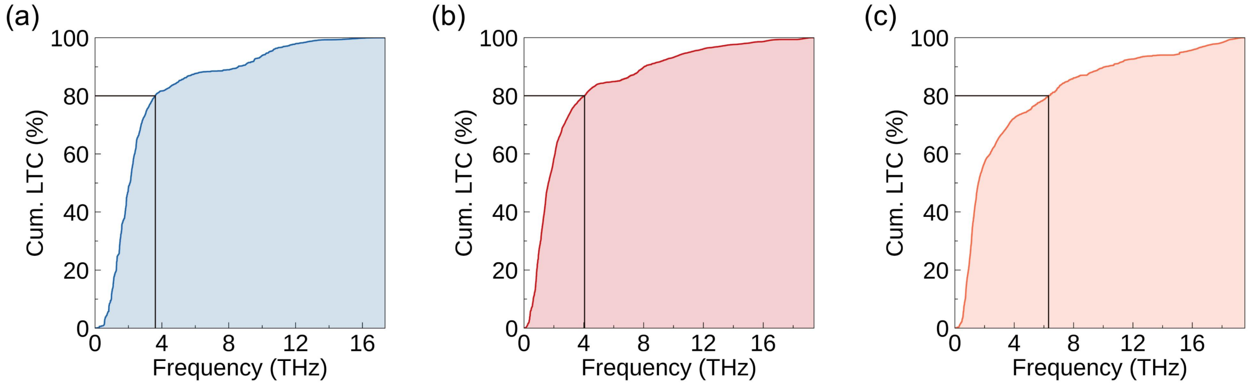

Figure S12 Cumulative lattice thermal conductivity as a function of phonon frequency at 300 K for (a) Bi_2YO_4Cl, (b) Bi_2LaO_4Cl, and (c) Bi_3O_4Cl. The horizontal line marks 80% of the cumulative lattice thermal conductivity, and the vertical line indicates the corresponding cutoff frequency.

11 Three-phonon scattering phase space

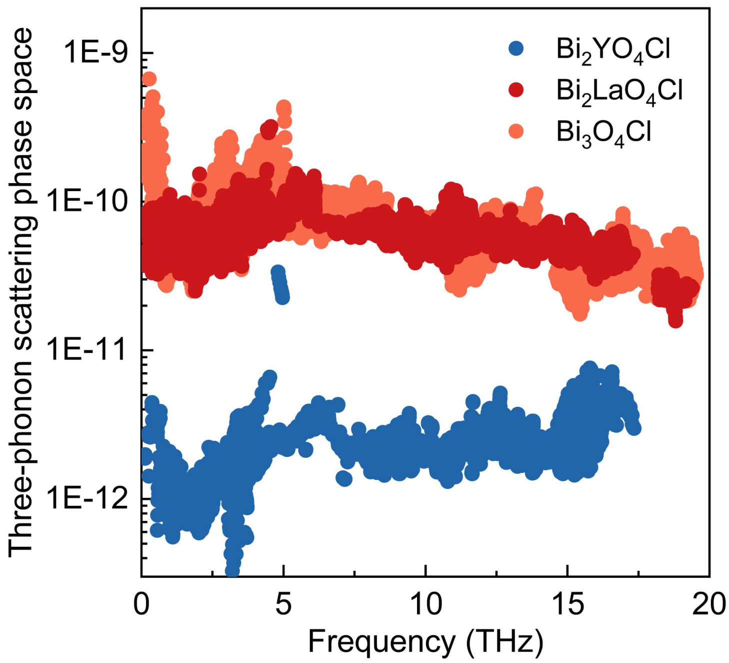

Figure S13 Three-phonon scattering phase space as a function of phonon frequency for Bi_2MO_4Cl (M = Y, La, and Bi).

References

1 A. Nakada, D. Kato, R. Nelson, H. Takahira, M. Yabuuchi, M. Higashi, H. Suzuki, M. Kirsanova, N. Kakudou, C. Tassel, T. Yamamoto, C. M. Brown, R. Dronskowski, A. Saeki, A. Abakumov, H. Kageyama and R. Abe, *J. Am. Chem. Soc.*, 2021, **143**, 2491-2499.

2 K. B. Spooner, M. Einhorn, D. W. Davies and D. O. Scanlon, *J. Open Source Softw.*, 2024, **9**, 6340.

3 C. J. Bradley and A. P. Cracknell, *The Mathematical Theory of Symmetry in Solids: Representation Theory for Point Groups and Space Groups*, Clarendon Press, Oxford, 1972.

4 Y. Zhang, E. Skoug, J. Cain, V. Ozoliņš, D. Morelli and C. Wolverton, *Phys. Rev. B*, 2012, **85**, 054306.

5 R. Hanus, J. George, M. Wood, A. Bonkowski, Y. Cheng, D. L. Abernathy, M. E. Manley, G. Hautier, G. J. Snyder and R. l. P. Hermann, *Mater. Today Phys.*, 2021, **18**, 100344.

6 R. Gurunathan, R. Hanus, M. Dylla, A. Katre and G. J. Snyder, *Phys. Rev. Appl.*, 2020, **13**, 034011.
